# The Self and Its Intersubjective Synchrony in Psychotherapy: A Systematic Review

**DOI:** 10.1002/cpp.70110

**Published:** 2025-07-01

**Authors:** Lorenzo Lucherini Angeletti, Bianca Ventura, Ferdinando Galassi, Giovanni Castellini, Valdo Ricca, Andrea Scalabrini, Georg Northoff

**Affiliations:** ^1^ Psychiatry Unit, Department of Health Sciences University of Florence Florence Italy; ^2^ The Royal's Institute of Mental Health Research & University of Ottawa Ottawa Ontario Canada; ^3^ Centro di Terapia Cognitivo Comportamentale (CTCC) Florence Italy; ^4^ Department of Human and Social Sciences University of Bergamo Bergamo Italy

**Keywords:** intersubjective, psychotherapy, reorganization, self, synchrony

## Abstract

The self is pivotal in psychotherapy, integrating internal (interoexteroceptive) and external (exteroproprioceptive) layers of experience necessary to create an intersubjective space in psychotherapeutic relationship to foster self‐reorganization. Patient–therapist intersubjective experience of synchrony seems to be core in better alliance and connectedness, favouring therapeutic progresses. However, the biobehavioural aspects of intersubjective synchrony are often disconnected from the self, leaving gaps in understanding its role in shaping therapy processes and outcomes. Following PRISMA guidelines, 32 studies measuring intersubjective synchrony during psychotherapy sessions were identified, covering interoexteroceptive (*n* = 11), exteroproprioceptive (*n* = 18) and interbrain (*n* = 4) synchrony. Exteroproprioceptive synchrony, particularly nonverbal, was primarily associated with the therapeutic alliance: This was further supported by interbrain studies, whereas interoexteroceptive synchrony was mainly linked to moment‐to‐moment emotional experience. On the other hand, we found mixed findings on the associations of exteroproprioceptive synchrony with overall therapy outcomes, whereas interoexteroceptive synchrony seemed to be positively associated with session‐level outcomes. Moreover, pre‐session intra‐subjective traits (e.g., patients' well‐being and psychopathology, therapists' attachment and expertise) influenced intersubjective synchrony patterns. Finally, we found that degrees of synchrony varied across patients suggesting different experiences of optimal psychotherapeutic relationship. Findings highlighted the layered dynamics of synchrony in psychotherapy. Achieving effective therapeutic engagement involves balancing context‐sensitive synchrony at different layers through intersubjective interaction for a self‐reorganization of the patients' dynamics. Future research should further explore how distinct layers and dynamics interact both at intrasubjective and intersubjective levels. A spatiotemporally informed emphasis on self‐related dynamics can illuminate common therapeutic factors and clarify their role in short‐ and long‐term outcomes.

**Summary:**

- The self is a dynamic, multilayered system that underlies coregulation processes within the therapeutic relationship. In this context, intersubjective synchrony reflects the encounter of self‐dynamics between patient and therapist, fostering alliance and emotional resonance.
- Physiological synchrony (interoexteroceptive) supports moment‐to‐moment affective attunement, whereas behavioural synchrony (exteroproprioceptive) sustains long‐term relational engagement.
- Therapist‐led nonverbal synchrony is particularly associated with stronger therapeutic alliance and better outcomes.
- Presession intrapsychic traits—such as patient well‐being or therapist expertise and attachment—modulate synchrony patterns and therapeutic responsiveness.
- Intersubjective synchrony offers a promising marker for precision psychotherapy, guiding self‐reorganization through tailored relational dynamics.

*If there is a synchronization between therapist and patient on the same kind of wavelength, no matter the kind of psychotherapeutic orientation, a kind of synchronization is engendered that produces the emotions necessary for reorganization. That is the basic core in common for everyone*.

(Guidano 1991, 204)

## 1 Introduction: The Self as a Dynamic and Relational Process in Psychotherapy

When a patient and a psychotherapist engage in a therapeutic encounter, each brings into the interaction their own behaviors, motivations, affective states and relational patterns—that is, the dynamic organization of their respective selves—contributing to a shared relational field. This intersubjective space becomes the context in which the therapeutic process unfolds. Within this field, the reciprocal interplay of the therapist's and patient's self‐dynamics provides the conditions for the patient's self to undergo gradual reorganization (Northoff 2013, 2016; Sui and Humphreys 2015).

The self can be conceptualized as a complex, dynamic system that sustains coherence by continuously integrating internal signals (e.g., heart rate, respiration and visceral states) with external inputs from the environment (e.g., facial expressions and social cues) (Craig 2009; Damasio 2010). When this integration functions optimally, it supports homeostasis, adaptive behaviour and a stable sense of identity. In contrast, disruptions in this integrative capacity—referred to as ‘basic disturbances of the self’ (Northoff 2014)—are evident across a range of psychiatric conditions, including mood, anxiety, trauma‐related, eating and neurodevelopmental disorders. In such cases, symptoms may be understood as maladaptive attempts to reestablish interoceptive–exteroceptive equilibrium in the face of impaired self‐organization (Lucherini Angeletti 2022, 2023; Lian and Northoff 2021; Scalabrini et al. 2020, 2022; Keskin et al. 2023; Sabbah and Northoff 2024).

Within this framework, psychotherapy can be viewed as an intervention aimed at facilitating the reorganization of the self of the patient through a structured intersubjective relationship. The therapeutic dyad provides a safe and bounded environment—a microcosm of self–other interactions—where maladaptive patterns can be expressed, examined and gradually transformed (Schore 2019). This process is grounded in the relational nature of the self, which is shaped and modulated through interpersonal dynamics from early development onward (Feldman 2017). By centring on these self‐related and relational processes, psychotherapy targets the core disruptions underlying psychopathology and supports the restoration of psychological integration and adaptive functioning.

In essence, a deep understanding of the self is crucial for improving psychotherapeutic processes, as the self underlies both mental and embodied experience and serves as the primary target of therapeutic change (see Box 1). It is therefore important to integrate contemporary perspectives on self‐organization and self‐processing to elucidate how the self operates across multiple levels of experience and how it can be modulated in therapy.

Box 1 **Conceptual foundations of the self in psychotherapy.**

The concept of self represents a fundamental psychological and biological property of complex living systems, characterized by their ability to interact with their environment in a self‐referential manner (Maturana and Varela 1980, 1984). This means that organisms constantly process information from their surroundings in relation to their own state and needs. In this context, all activities of such a system aim to maintain its inner stability (or homeostasis) and thus its conservation within a constantly changing outer environment. This principle extends to human experience, where the dynamic integration of external environmental information with internal states and needs facilitates the development of a stable and coherent *self‐organization*. The latter refers to the process by which a system spontaneously arranges its components to maintain stability and functionality without external directions (Camazine et al. 2003), thereby promoting adaptive functioning and self‐preservation. Central to this self‐organization is the continuous interplay between interoceptive signals (internal bodily sensations) and exteroceptive stimuli (information from the external environment). This ongoing integration forms the foundation for the brain's self‐organizing activities, enabling the system to maintain stability while flexibly adapting to changing circumstances (Craig 2009; Damasio 2010).

Moreover, psychological and psychotherapeutic research over the years has consistently highlighted the self's inherently relational nature. Bowlby (1969, 1988) revolutionized our understanding of the self by demonstrating that its development relies on early intersubjective interactions, where an infant's emotional activity and reactivity are shaped by the spatial and temporal dynamics of a caregiver's responses. Building on Bowlby's attachment theory, numerous cognitive and psychodynamic authors have elaborated complex models of the self, examining how intersubjective exchanges—particularly those between caregiver and infant—significantly shape the developing self (Guidano and Liotti 1983; Guidano 1987, 1991; Reda 1991; Schore 2000, 2001a, 2001b, 2012; Lyons‐Ruth 2003, 2008; Fonagy and Bateman 2007; Mucci 2013, 2018; Beebe and Lachmann 2020).

Similarly, several major psychotherapeutic approaches—both cognitive and psychodynamic—have placed the self, its development and its organization at the centre of their theoretical frameworks. These perspectives view the self as a dynamic system that crucially influences an individual's physical, emotional and cognitive experiences. They also emphasize the self's fundamentally relational nature, defined and continuously reshaped by intersubjective relationships from the infant–caregiver bond to the patient–therapist interaction (Kohut 1957; Sullivan 1953; Kelly 1955; Guidano 2009; Bromberg 2009; Beebe and Lachmann 2020).

Understanding the dynamics and organization of the self has long been a cornerstone of psychotherapeutic theory and practice, and it continues to serve as a central focus for advancing empirical research on therapeutic mechanisms of change.

Recent neuroscientific work has further enriched this conceptualization by identifying distinct yet interconnected layers of self‐related processing (Damasio 2010; Panksepp 1998; Seth 2013 ). Qin et al. (2020) proposed a hierarchical model comprising three nested layers: the *interoexteroceptive self*, the *exteroproprioceptive self* and a higher order *mental self*. The present systematic review focuses on the first two layers, which constitute the prereflective levels most relevant to patient–therapist interaction in the here‐and‐now of the session.

The *interoexteroceptive self* refers to the integration of internal bodily signals—such as heart rate, respiration and visceral states—with basic external sensory input, forming the foundation for emotion and bodily awareness (Craig 2009; Damasio 2010; Babo‐Rebelo et al. 2016; Park and Blanke 2019). The *exteroproprioceptive self* builds on this by linking multisensory perception (e.g., vision and touch) with proprioceptive feedback from movement, enabling orientation, agency and interaction with the environment (Sperduti et al. 2011; Tsakiris 2017). Together, these layers underpin bodily self‐consciousness, body ownership and the first‐person perspective and are crucial for maintaining coherent self–other boundaries (Suzuki et al. 2013; Park and Blanke 2019). These two layers are hierarchically embedded within one another, also involving distinct neural systems: the interoexteroceptive layer engages subcortical regions as well as insula, operating on faster timescales, whereas the exteroproprioceptive layer recruits broader cortical areas like the premotor cortex and temporo‐parietal junction, associated with slightly slower dynamics (Qin et al. 2020; Northoff et al. 2020a, 2020b). When this multilayered organization is disrupted—particularly through relational trauma—it may lead to emotional dysregulation, fragmented identity and impaired interpersonal functioning (Scalabrini et al. 2022; van der Kolk 2014). Psychotherapy can thus be understood as a context in which these disrupted layers of the self are reintegrated, promoting more adaptive self‐organization and intersubjective coherence (Northoff and Scalabrini 2021).

Beyond the structural facets of the self, it is also essential to consider its dynamics within the therapeutic dyad. Psychotherapy may be viewed as a complex dynamic system comprising two interacting self‐organizing agents—the patient and the therapist—each bringing their own multilayered self into the encounter (Gelo and Salvatore 2016). Within this system, one can distinguish *intrasubjective dynamics*, which are the internal coordination and regulation between the layers of the self within each individual (Bloch et al. 2019), and *intersubjective dynamics*, which are the processes of interaction and attunement between patient and therapist (Fuchs and De 2009; Schore 2021). The degree of attunement achieved at this intersubjective level—in effect, the harmonization of the two persons' psychological, physiological and behavioural processes—has a profound influence on the therapeutic alliance and the efficacy of the treatment.

**FIGURE 1:**
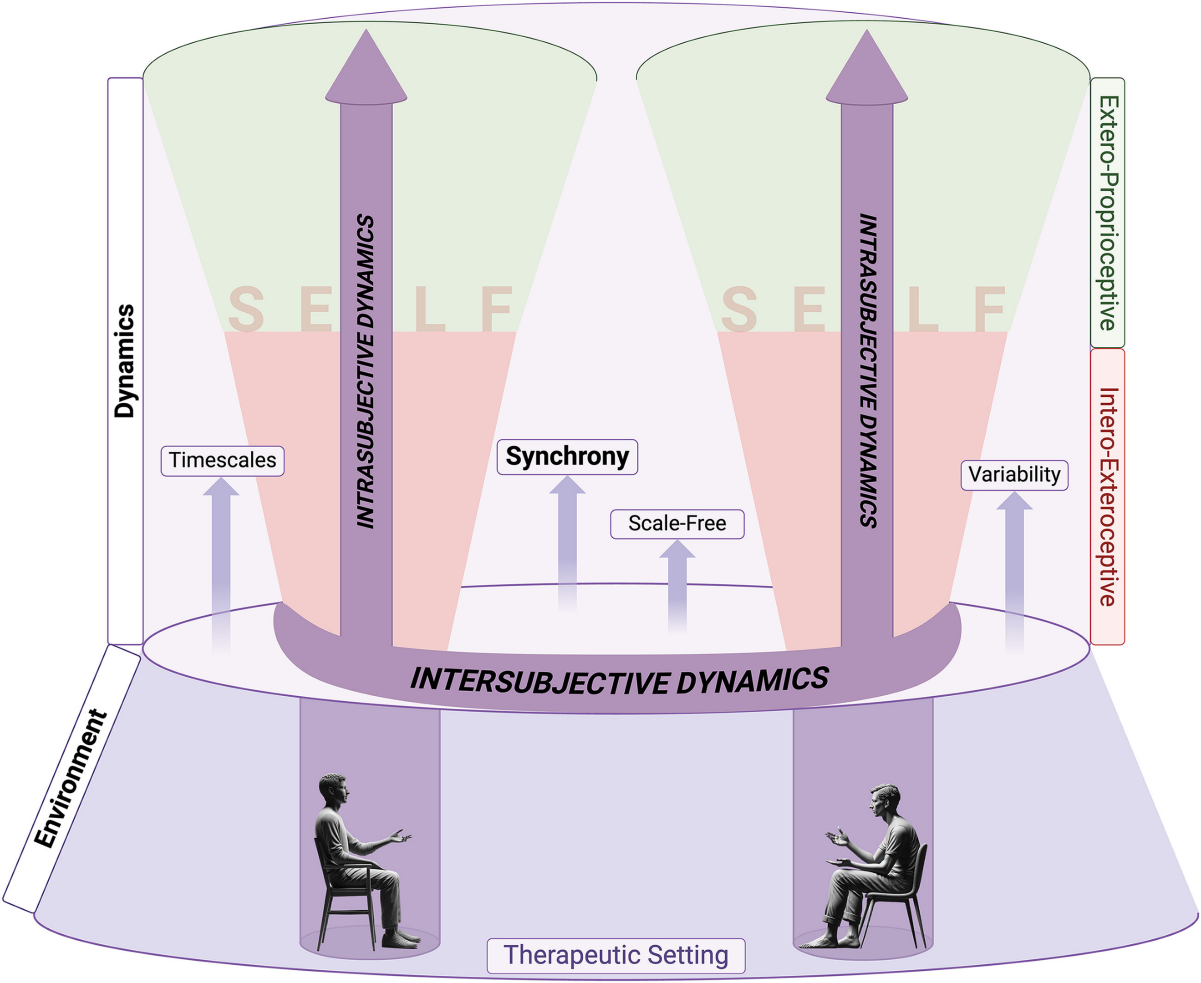
Visual representation of the self and its dynamics in psychotherapy. The figure illustrates the interplay between intrasubjective and intersubjective processes within the therapeutic setting. The *interoexteroceptive self* processes internal physiological signals (cardiac, respiratory and electrodermal activity), whereas the *exteroproprioceptive self* focuses on external stimuli (such as prosodic and nonverbal cues) representing the expression/manifestation of both layers in the therapeutic setting. *Intrasubjective dynamics* refer to internal processes within each individual's self, encompassing both interoexteroceptive and exteroproprioceptive layers and their interaction. *Intersubjective dynamics* represent the interactions and mutual influences between the therapist's and patient's layers of self. Key dynamic elements such as synchrony, timescales, scale‐freeness and variability are shown operating across both intrasubjective and intersubjective domains. Created with BioRender.com.

In recent years, a growing body of psychotherapy research has focused on the phenomenon of *synchrony* to capture and quantify these complex dynamics (Koole and Tschacher 2016; Palumbo et al. 2017; Kleinbub 2017; Wiltshire et al. 2020). Broadly defined, synchrony refers to the coordinated fluctuations of physiological or behavioural signals between two individuals that unfold in a temporally aligned manner (Koole and Tschacher 2016). In the therapeutic context, it serves as an index of moment‐to‐moment attunement between patient and therapist. Importantly, synchrony is not a process exclusive to therapy; it represents a fundamental form of coordination observed across living systems (Pikovsky et al. 2001) and has been described as a *common currency* linking biological, affective and social levels of interaction (Northoff et al. 2020a, 2020b; Northoff 2024; Scalabrini et al. 2022). By examining synchrony within psychotherapy, we can thus bridge the theoretical understanding of self‐related dynamics with empirical observations of how patient and therapist engage in a shared rhythm during the therapeutic process (Figure 1).

Building on this theoretical framework, the following section delves into the construct of intersubjective synchrony between patient and therapist, which lies at the heart of our systematic review.

### 1.1 Intersubjective Synchrony Between the Layers of Self in Psychotherapy

The focus on synchrony results from neuroscientific and psychological research highlighting the importance of synchronization processes in intersubjective functioning. Herein, *intersubjective synchrony* represents a multifaceted and complex process whereby individuals resonate with each other's emotions, intentions and actions (Fuchs and De 2009; Palumbo et al. 2017; Schore 2021). This form of relational synchrony appears to have deep evolutionary roots, reflecting the biological imperative of human existence which inherently compels us to be relational beings to survive (Feldman 2017). Moreover, it encompasses four specific functions: simplifying interactions (by increasing predictability), facilitating coordinated task performance, strengthening social bonds and enabling individuals to influence each other's behavior (daSilva and Wood 2024). For instance, when people form connections or coexist in shared spaces, they often exhibit a natural tendency to synchronize their neural activity (Hasson et al. 2004), physiological responses (Ferrer and Helm 2013) and behaviours with those around them (Ramseyer and Tschacher 2011). This tendency towards synchronization has been considered a basic relational process with positive effects on engagement (Murata et al. 2021), affiliation (Hove and Risen 2009), attraction (Prochazkova et al. 2022), performance (Gordon et al. 2020), tendency to cooperate (Behrens et al. 2020) and prosocial behaviours and attitudes (Rennung and Göritz 2016).

The integration of these insights is essential in understanding the dynamics between patient and therapist in psychotherapy. A central reason patients enter therapy lies in their difficulty sustaining intersubjective attunement and regulating their internal states—difficulties that often manifest across behavioral, emotional and physiological domains. For instance, individuals with early attachment disruptions may show impaired bodily self‐awareness, limited affective mirroring or dysregulated physiological responses (Lucherini Angeletti et al. 2023, 2022; Lian and Northoff 2021; Scalabrini et al. 2022; Keskin et al. 2023; Sabbah and Northoff 2024). These disruptions may lead to initial difficulties in establishing synchrony with the therapist. In line with the neurobiology of human attachment (Feldman 2017), this dissynchrony reflects longstanding intrasubjective and intersubjective impairments that are precisely what psychotherapy seeks to address. Over time, however, the therapeutic relationship may offer a new relational context where the patient can progressively experience more regulated and attuned interaction patterns. In this sense, synchrony is not only a marker of therapeutic success but also a means of restoring self‐organization through embodied coregulation. Yet previous neurobiological and behavioral research in psychotherapy has rarely addressed how these synchrony dynamics are rooted in the disrupted organization of the self.

**FIGURE 2:**
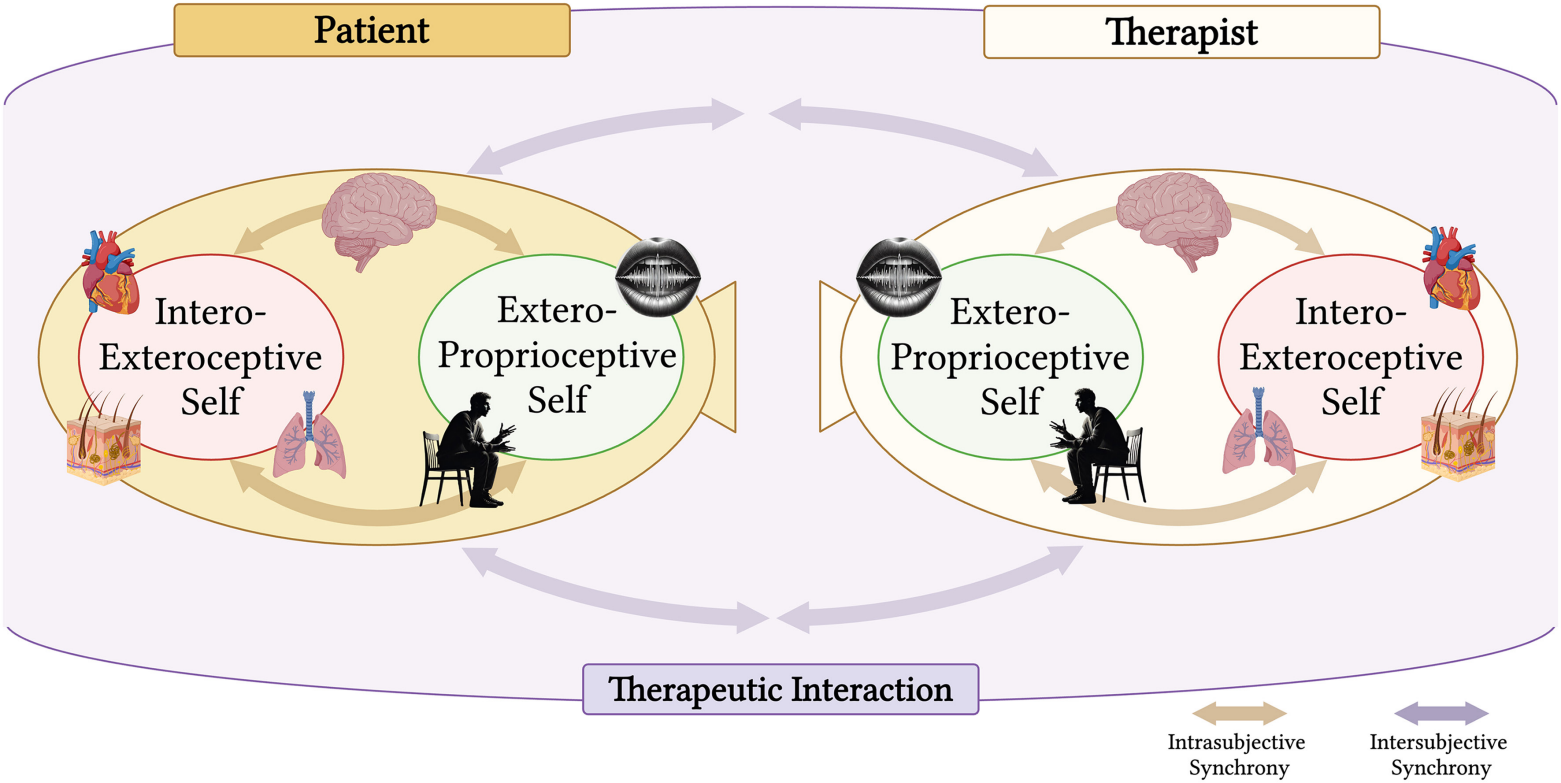
Visual representation of the interaction between the *interoexteroceptive* and *exteroproprioceptive* selves of the patient and therapist within the therapeutic interaction. The *interoexteroceptive self* processes internal physiological signals (cardiac, respiratory and electrodermal activity), whereas the *exteroproprioceptive self* focuses on external stimuli (such as prosodic and nonverbal cues) representing the expression/manifestation of both layers in the therapeutic setting. *Intrasubjective synchrony* (i.e., internal coordination between these layers) is shown within each individual (yellow arrows), whereas *intersubjective synchrony* (i.e., the reciprocal influence between patient and therapist) supports the therapeutic interaction (purple arrows). This dynamic interplay fosters a shared therapeutic space. Created with BioRender.com.

In line with a view that places the self and its dynamics at the core of the psychotherapeutic process, intersubjective synchrony can manifest across the different layers of the self. Given the nested hierarchy of these layers, we can hypothesize that synchrony at the interoexteroceptive layer of the self may correspond to physiological synchrony (e.g., heart rate, breathing patterns and electrodermal activity/EDA), which has been widely discussed in both clinical and nonclinical literature (for a review, see Palumbo et al. 2017). Concrete manifestations of this synchrony include, for example, moments of emotional attunement in psychotherapy during which the patient and therapist may prereflexively align their breathing rhythms or experience concurrent fluctuations in heart rate and skin conductance while discussing emotionally salient content (Marci et al. 2007; Tschacher and Meier 2019). On the other hand, synchrony at the exteroproprioceptive layer—referring to the dynamic alignment between multisensory perception of the external environment and proprioceptive feedback from one's bodily movements, posture or expressions—may reflect prosodic (e.g., tone of voice and speech rhythm) and nonverbal (e.g., gestures, body posture and facial expressions) synchrony, which has already been explored in psychotherapy research (for a review, see Koole and Tschacher 2016). For instance, the therapist and patient may gradually converge in posture, lean angle or microexpressions during an emotionally charged session, promoting empathic resonance and nonverbal mutual understanding (Ramseyer and Tschacher 2011). Furthermore, based on the specific dynamics of these two layers, we can hypothesize that each layer may naturally tend to synchronize more easily with the dynamics of its counterpart in the other person involved in the interaction (i.e., the patient's interoexteroceptive self with the therapist's interoexteroceptive self and the patient's exteroproprioceptive self with the therapist's exteroproprioceptive self) (Figure 2). This alignment between the layers of both patient's and therapist's self may play a crucial role in shaping therapeutic processes and predicting therapeutic outcomes.

How the patients' interaction with the therapist can foster an intrasubjective and intersubjective reorganization of the patient's self remains yet unclear. In addition, the neuropsychophysiological dynamics underlying this process are not clear yet either. Addressing these questions is relevant to possibly determine markers, both psychological and biological, that index the dyad's predisposition or propensity for synchrony and its possible psychotherapeutic responsivity. These questions are significant because such markers could potentially be used to personalize the psychotherapeutic process and tailor it to each individual's needs. This personalized approach could enhance the effectiveness of psychotherapy by aligning the therapeutic techniques with the patient's inherent ability to synchronize with the therapist, ultimately leading to more successful therapeutic processes and outcomes and a deeper understanding of the therapeutic relationship dynamics.

### 1.2 Aims of the Systematic Review

To address these yet open issues, the current work aims to provide a systematic review of studies that have investigated intersubjective synchrony in psychotherapy. In detail, departing from the theoretical framework of the hierarchical and dynamical model of the self and its different layers (Qin et al. 2020), we sought to disentangle the different types of intersubjective synchrony and their associations with specific therapeutic processes and outcomes. We focused on research investigating the simultaneous recording (in both patient and therapist) of indexes related specifically to the processing of the interoexteroceptive self (such as heart rate, breathing patterns and EDA) and indexes connected to the processing of the exteroproprioceptive self (including prosodic aspects and nonverbal behaviour). Within the therapeutic exchange, these two layers can be viewed as distinct components each with its own dynamics, reciprocally operating within and between patient and therapist (Figure 2).

Additionally, we included studies on *interbrain synchrony* as illustrative examples of how intersubjective synchrony can extend to the neural level, reflecting shared brain dynamics during therapeutic interaction. While still an emerging field, such studies offer promising insights into how the alignment of neural oscillations—particularly during moments of affective resonance or shared understanding—may support relational attunement and contribute to deeper therapeutic connection across self layers. This comprehensive approach allowed us to explore the multilayered dynamics of synchrony of both patient's and therapist's selves within the therapeutic interaction. Furthermore, because synchrony is a multifaceted process that can be studied with different approaches, we summarized the analysis methods used in the reviewed studies (Box 2). Finally, presupposing a spatiotemporal approach as our umbrella framework (Northoff & Scalabrini 2021; Northoff 2024), we proposed a dynamic perspective of intersubjectivity in psychotherapy that can address the specific dynamics underlying therapeutic processes and outcomes.

Box 2 **Analytical methods for measuring synchrony in psychotherapy.**

The analysis of synchrony in the reviewed studies has employed a diverse range of methods, each tailored to capture different aspects of intersubjective processes.

Cross‐correlation techniques formed the backbone of many studies, with variations adapted to specific research contexts. *Cross‐correlation within defined time windows* was widely used across both interoexteroceptive (Marci et al. 2007; Messina et al.^2013^; Karvonen et al. 2016; Tourunen et al. 2020; Coutinho et al. 2023; Bar‐Kalifa et al. 2023; Gernert et al. 2023) and exteroproprioceptive synchrony studies (Ramseyer and Tschacher 2011, 2014; Reich et al. 2014; Rocco et al. 2018; Paulick et al. 2018a, 2018b; Ramseyer 2019; Prinz et al. 2020; Zimmermann et al. 2021; Deres‐Cohen et al. 2021a), allowing researchers to detect moment‐to‐moment alignment in physiological and behavioral data. Indeed, segmenting biobehavioural data into specific windows (e.g., 5‐ to15‐s intervals) allowed researchers to calculate correlations within those intervals and detect alignment over brief, well‐defined periods.

*Cross‐correlation functions* (CCFs) extended this approach to analyse synchrony over longer periods in both interoexteroceptive (Bar‐Kalifa et al. 2019; Prinz et al. 2021) and exteroproprioceptive synchrony studies (Galbusera et al. 2016), providing insights into lead–lag relationships between therapist and client responses. CCFs offer a broader view of how synchrony unfolds continuously over time and are particularly useful in understanding long‐term dynamics of physiological and behavioural alignment.

*Windowed cross‐lagged correlation* (WCLC), used in exteroproprioceptive studies (Altmann et al. 2019; Schoenherr et al. 2019; Lutz et al. 2020; Gernert et al. 2023), offers an analysis of nonverbal synchrony, evaluating how movements align over time and determining leader–follower dynamics. In two of these studies, the *peak‐picking algorithm* was used with WCLC to detect key moments of synchrony (Schoenherr et al. 2019; Lutz et al. 2020). It identifies peaks in the cross‐correlation function within a ± 5‐s time lag, focusing on significant intervals while excluding those shorter than 0.4 s to ensure meaningful synchrony is captured, filtering out random noise.

To enhance the robustness of these analyses, *surrogate synchrony* (SUSY) and *surrogate concordance* (SUCO) methods were introduced, using surrogate data to validate observed synchrony statistically. These methods, applied in both interoexteroceptive (Tschacher and Meier 2019; Coutinho et al. 2023) and exteroproprioceptive studies (Nyman‐Salonen et al. 2021), ensured that the detected synchrony reflects genuine interaction rather than chance.

The *concordance index* (CI) method, used specifically for EDA synchrony, offered a way to quantify physiological synchronization and its relation to empathy and emotional engagement (Marci et al. 2007; Palmieri et al. 2018a). Specifically, it has been calculated by correlating the slopes of EDA levels over successive 5‐s windows. Pearson correlations were computed within 15‐s intervals, and the ratio of positive to negative correlations determined the final index. A higher CI indicated stronger physiological synchronization, reflecting attunement during therapy sessions.

Additionally, *lag analysis* was performed to assess whether one party led the synchrony or followed it (Palmieri et al. 2018a). This method introduced time lags (e.g., ± 5 s) to evaluate whether the therapist's or the patient's physiological signal was leading the synchrony. In cases where synchrony was higher at negative lags, the therapist's responses were leading the interaction, whereas positive lags indicated that the patient was leading. Notably, some studies ventured beyond linear analyses, with one exteroproprioceptive study employing polynomial regression and response surface analysis to explore non‐linear relationships in synchrony data (Deres‐Cohen et al. 2021b).

In the realm of interbrain synchrony, more specialized techniques were employed. A study employed *phase locking value* (PLV) to measure interbrain synchrony between patient and therapist wearing dual‐EEG (Lecchi et al. 2020). This method of analysis calculated the degree to which brainwave oscillations at specific frequencies (Theta and Alpha bands) were synchronized across time. It measured the consistency of phase differences between EEG signals from therapist–patient dyads, indicating how ‘in sync’ their brain activity was during therapy sessions. PLV assessed synchronization in frontal and central brain regions during therapy sessions, identifying moments of neural coupling.

*Wavelet transform coherence* (WTC) was used to measure neural synchrony between therapists and patients by analysing the synchrony of oxygenated haemoglobin levels during hyperscanning (Zhang et al. 2018, 2019). After filtering global signals with principal component analysis, WTC assessed brainwave coherence over time. This method aligns with cross‐correlation techniques used in other synchrony studies, allowing potential aggregation.

Finally, *cross‐spectral analysis* was applied to measure interbrain synchronization (Akimoto et al. 2021). This method involved calculating the cross‐spectrum of hyperscanning signals between homologous brain regions of therapists and patients. They tracked changes in oxygenated haemoglobin in the PFC and FP regions, focusing on low‐frequency oscillations (0.01–0.08 Hz) to identify synchrony between therapists and patients during sessions.

Although primarily developed for intersubjective analysis, these methods could be adapted to evaluate coordination among different physiological, behavioural and neural systems within an individual. For example, cross‐correlation and surrogate synchrony methods could assess interactions between brain, heart, lungs, EDA and nonverbal behaviour, providing insights into intrasubjective coherence and self‐organization before and during the psychotherapeutic course. This underscores the interconnected nature of intersubjective and intrasubjective processes in psychotherapy research, which needs to be further investigated.

## 2 Materials and Methods

### 2.1 Eligibility Criteria and Information Sources

This systematic review followed the PRISMA guidelines to ensure transparent and comprehensive reporting. The eligibility criteria for the inclusion and exclusion of studies were established based on predefined parameters to ensure that only relevant, high‐quality studies were considered. The search was conducted using *PubMed* and *Scopus*.

Inclusion criteria for this systematic review were carefully designed to ensure a focused and comprehensive analysis. Studies were required to be quantitative in nature and centred on psychotherapy. A crucial requirement was the simultaneous measurement of synchrony during therapy sessions, capturing real‐time dynamics within the therapeutic context. Furthermore, studies needed to employ dyadic‐level data, specifically measuring synchrony between therapist and patient or within therapeutic tetrads (i.e., couple therapy). Lastly, to be included, studies had to investigate both the therapeutic process and outcome, enabling an exploration of how synchrony relates to the overall effectiveness of psychotherapy. We omitted studies lacking simultaneous, dyadic synchrony measurements during therapy sessions and those using only self‐report or nontherapeutic synchrony measures. Qualitative studies, case studies and those with fewer than three dyads were also excluded. By adhering to these criteria, we ensured a rigorous selection of studies that could provide meaningful insights into the role of synchrony in psychotherapeutic interactions and outcomes. Finally, studies were grouped for synthesis based on their approach to synchrony measurement, therapeutic context and type of outcomes assessed. Specifically, studies were categorized by synchrony measurement indices (e.g., physiological, prosodic/nonverbal or neural) and whether they focused on process‐related or outcome‐related variables.

### 2.2 Search Strategy and Selection Process

**FIGURE 3:**
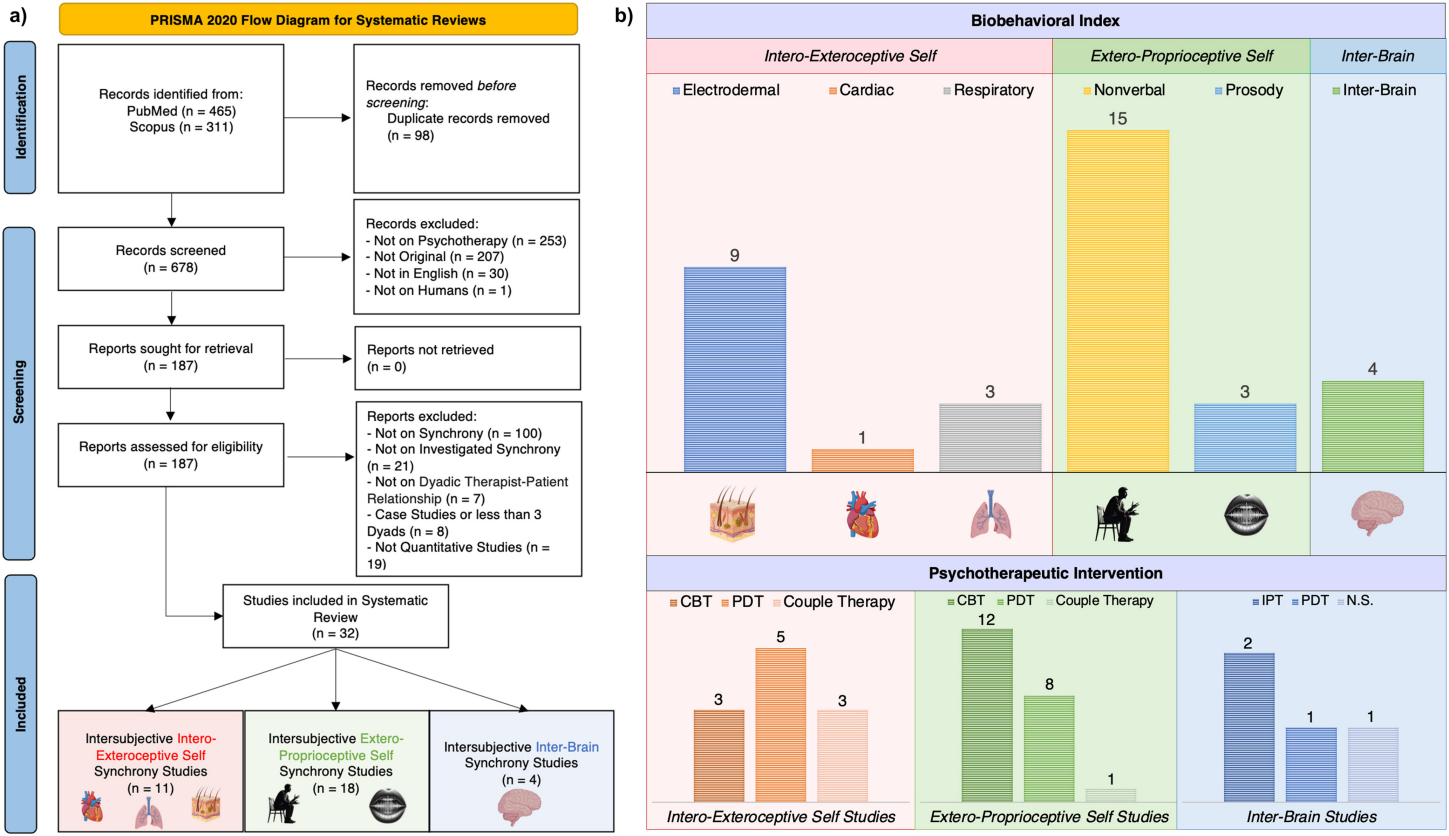
(a) PRISMA flowchart of the article selection process. (b) Graphic illustration of the examined studies, which were divided into *interoexteroceptive self*, *exteroproprioceptive self* and *interbrain* indexes. The lower section shows the distribution by *psychotherapeutic interventions* in the different layers. Abbreviations: CBT, cognitive‐behavioural therapy; NS, not specified; PDT, psychodynamic therapy.

To ensure the inclusion of studies of comparable quality, only those in English and published in scientific journals were considered for this work. The systematic literature search and study selection process adhered to PRISMA guidelines (Figure 3a). Information was sourced from PubMed and Scopus, with an online search conducted using the following query: (*psychotherapy*) AND (*synchrony OR synchronization OR coordination OR attunement*) AND (*interpersonal OR intersubjective OR dyadic OR therapeutic relationship OR therapeutic alliance OR intrapersonal OR intrasubjective*) NOT (‘*review*’[*pt*] OR ‘*systematic review*’[*pt*] OR ‘*meta‐analysis*’[*pt*]) for the time frame up to March 2025. To complete the search strategy, once the eligible studies were identified, we reviewed the references of these studies to find other studies eligible for review. Finally, we went over reviews and meta‐analyses already in the literature to find additional studies that we could have left out (Koole and Tschacher 2016; Palumbo et al. 2017; Kleinbub 2017; Wiltshire et al. 2020).

### 2.3 Data Collection Process and Items

Data collection for this systematic review was conducted by two independent reviewers (LLA and BV). Each reviewer independently extracted data from the selected studies, focusing on specific variables of interest, including the number of subjects involved (patients and therapists), patient sample characteristics, the type of psychotherapy used, the therapeutic domain investigated (processes or outcomes), the used index, the method of analysis for measuring synchrony and the study's key findings (Tables 1, 2, 3). After completing the independent data extraction, the two reviewers compared their findings to ensure accuracy and completeness. Any discrepancies or missing information was discussed and resolved through consensus. No automation tools were used, and there was no need to contact study investigators for additional data.

**TABLE 1:** Summary of intersubjective interoexteroceptive self synchrony studies.

Study	Sample	Patient sample features	Intervention	Therapeutic domain		I‐E Index	Analysis	Findings
				Process	Outcome			
1. Marci et al. (2007)	20 dyads	AffD AnxD	PDT	Perceived therapist empathy	—	EDA	Concordance Index	Higher EDA synchrony associated with higher Pt′s‐perceived Th empathy and more positive social–emotional interactions during therapy
2. Messina et al. (2013)	39 dyads	—	PDT (simulated clinical interviews)	Perceived therapist empathy	—	EDA	Cross‐correlations within defined time windows	Higher levels of EDA synchrony associated with higher Pt′s‐perceived Th empathy
3. Karvonen et al. (2016)	20 Pt (10 couples) 10 Th	—	Couple therapy	Sympathetic nervous system synchrony	Session‐level outcomes	EDA	Cross‐correlations within defined time windows	Highest SNS synchrony observed between co‐Ths, followed by Pt‐Th dyads, and the lowest synchrony between Pt‐Pt SNS synchrony associated with Pt′s and Th′s session‐level evaluation
4. Palmieri et al. (2018a)	18 dyads	—	PDT (simulated clinical interviews)	Effect of Ths' attachment‐security priming and positive affect on synchrony	—	EDA	Concordance Index and lag analysis	Attachment‐security priming resulted in Ths *leading* EDA synchrony, with higher EDA synchrony observed when Ths anticipated Pts' responses Positive‐affect priming resulted in Ths *following* EDA synchrony, with higher EDA synchrony observed while following their Pts' activity No significant difference in overall synchronization between the attachment‐security and positive‐affect conditions
5. Tschacher and Meier (2019)	4 dyads	—	PDT	Therapeutic alliance	Session‐level outcomes	HR HRV ECG BR	Computation of ATIONS with SUSY Window‐wise slopes with SUCO	In‐phase respiration synchrony across SUCO and SUSY, with the latter associated with Pt′s alliance and Th′s progress ratings Antiphase HR synchrony associated to Th′s alliance ratings In‐phase HR synchrony associated to Th′s progress ratings In‐phase HRV synchrony associated to Th′s alliance Antiphase HRV synchrony associated to Th′s cooperation ratings No associations between synchrony and ECG
6. Bar‐Kalifa et al. (2019)	31 Pt 10 Th	Test Anxiety	CBT (with imagery‐based techniques)	Therapeutic alliance	—	EDA	CCFs with time‐series analysis	Higher EDA synchrony associated with higher Pt′s reported therapeutic alliance
7. Tourunen et al. (2020)	20 Pt (10 couples) 10 Th	—	Couple therapy	Therapeutic alliance	Overall therapy outcomes	EDA	Cross‐correlations within defined time windows	EDA synchrony between Pt‐Pt increased over the course of therapy Pts‐Ths EDA synchrony remained stable throughout the therapy sessions No significant associations between Pt‐Th EDA synchrony and therapeutic alliance or therapy outcomes
8. Prinz et al. (2021)	50 Dyads	Test anxiety	CBT (with imagery‐based techniques)	Session‐level intrasubjective Pts' emotional experiences	—	EDA	CCFs	Th‐led synchrony associated with Pt′s positive emotional experience (i.e., contentment, vigour and calmness) and with Pt′s lower negative emotional experience (i.e., anxiety, depression and fatigue)
9. Coutinho et al. (2023)	24 Pt (12 couples) 10 Th	—	Couple Therapy	Therapeutic alliance Effect of Pt′s before sessions well‐being on synchrony	—	EDA BR	Cross‐correlations within defined time windows SUSY	In‐phase EDA synchrony in Pt‐Th and Th‐Th dyads, both not associated with therapeutic alliance Anti‐phase EDA synchrony in Pt‐Pt dyad predicted by Th′s after sessions rated therapeutic alliance and Pt′s before sessions well‐being No significant synchrony for respiration
10. Bar‐Kalifa et al. (2023)	28 Pt 9 Th	MDD	Short‐term PDT	Session‐level intrasubjective Pts' emotional experiences	Session‐level outcomes	RSA	Cross‐correlations within defined time windows	Higher in‐phase and antiphase RSA synchrony during moments of clients' positive and productive emotional experiences, also linked to better session evaluations
11. Gernert et al. (2023)	25 dyads	Not specified diagnosis	CBT	—	Overall therapy outcomes	EDA	Single Session Index (SSI) algorithm	Higher EDA synchrony positively associated with higher symptom reductions Higher EDA synchrony negatively associated with symptom aggravation

Abbreviations: AffD, affective disorder; AnxD, anxiety disorder; BR, breathing rate; CBT, cognitive‐behavioural therapy; CCFs, cross‐correlation functions; ECG, electrocardiogram; EDA, electrodermal activity; HR, heart rate; HRV, heart rate variability; MDD, major depressive disorder; PDT, psychodynamic therapy; Pt, patient; RSA, respiratory sinus arrhythmia; SET, supportive‐expressive therapy; SNS, sympathetic nervous system; SSI, Single Session Index; SUCO, surrogate concordance; SUSY, surrogate synchrony; Th, therapist.

**TABLE 2:** Summary of intersubjective exteroproprioceptive self synchrony studies.

Study	Sample	Patient sample features	Intervention	Therapeutic domain		E‐P Index	Analysis	Findings
				Process	Outcome			
1. Ramseyer and Tschacher (2011)	70 dyads	AffD AnxD AdjD PersD	CBT	Therapeutic alliance	Overall therapy outcomes	Nonverbal (MEA)	Cross‐correlations within defined time windows	Higher synchrony associated with higher Pt′s therapeutic alliance rates Higher synchrony associated with greater symptoms reduction
2. Ramseyer and Tschacher (2014)	70 dyads	AffD AnxD AdjD PersD	CBT	Therapeutic alliance	Overall therapy outcomes	Nonverbal (MEA)	Cross‐correlations within defined time windows	Body‐synchrony linked to session‐level Pt′s therapeutic alliance Head‐synchrony associated with overall therapy outcome (i.e., goal attainment)
3. Reich et al. (2014)	52 Pt 16 Th	—	CBT	Therapeutic alliance	Overall therapy outcomes	Prosody (vocal pitch)	Cross‐correlations within defined time windows	Higher Th‐led pitch synchrony related to poorer Pt′s therapeutic alliance Higher Th‐following pitch synchrony associated to Pt′s greater depressive symptoms
4. Galbusera et al. (2016)	16 Pt Th n.s.	SCZ spectrum	PDT (BPT)	—	Overall therapy outcomes (i.e., negative symptoms)	Nonverbal (MEA)	CCFs	Significant increase in synchrony from beginning to end of therapy in the dyad Positive trend toward association between synchrony and better overall therapeutic outcomes (e.g., reduction in negative symptoms)
5. Rocco et al. (2018)	5 dyads	AnxD PersD	PDT	Effect of intrasubjective paraverbal communication on synchrony	—	Prosody (vocal speech rate)	Cross‐correlations within defined time windows	Increased Pt′s speech rates associated with a higher self‐relevant formal organization of discourse (i.e., clarity and specificity) Decreased Pt′s speech rates associated with a higher self‐relevant evocative or sensory imagery (i.e., concreteness and imagery) Th′s speech rate attunement to Pt′s decreased speech rates during self‐relevant evocative or sensory imagery
6. Paulick et al. (2018a)	93 Pt	AnxD MDD	CBT	Effect of depression and anxiety on synchrony	—	Nonverbal (MEA)	Cross‐correlations within defined time windows	Dyads with depressive Pts had lower levels of nonverbal synchrony at the beginning of therapy than dyads with anxious Pts Nonverbal synchrony of dyads with depressive Pts increased over the course of therapy, whereas that of dyads with anxious Pts decreased
7. Paulick et al. (2018b)	143 Pt 27 Th	AffD AnxD AdjD PersD ED	CBT	Therapeutic alliance	Overall therapy outcomes (i.e., drop outs)	Nonverbal (MEA)	Cross‐correlations within defined time windows	Synchrony at the beginning of treatment did not predict the therapeutic alliance Highest synchrony with Th among Pts with no improvement who had consensual termination Medium levels of synchrony with Th among Pts who showed improvements Lowest synchrony with Th among nonimproved Pts who dropped out, even controlling for therapeutic alliance
8. Ramseyer (2019)	12 Pt 10 Th	6 AffD 3 AdjD 1 ED 2 no diagnosis	CBT	Effect of symptom distress on synchrony Therapeutic alliance	Overall therapy outcomes (i.e., interpersonal problems) Session‐level outcomes	Nonverbal (MEA)	Cross‐correlations within defined time windows	Synchrony not associated with therapeutic alliance Higher Pt′s symptom distress at intake associated with lower synchrony across therapy Higher synchrony associated with lower interpersonal problems improvements Lower synchrony associated with higher interpersonal problems improvements Higher synchrony associated to Th′s lower evaluation of Pt′s progress ratings
9. Altmann et al. (2019)	267 Pt Th n.s.	SAD	CBT PDT	Therapeutic alliance	Overall therapy outcomes	Nonverbal (MEA)	WCLC	Higher synchrony in an early phase of therapy predicted a better therapeutic alliance at session 20 Higher Th‐led synchrony in an early phase of therapy predicted reduced interpersonal problems at the end of therapy Higher Pt‐led synchrony associated with higher depressive symptoms and interpersonal problems at the end of therapy
10. Schoenherr et al. (2019)	267 dyads (267 Pt 119 Th)	SAD	CBT PDT	—	Overall therapy outcomes (i.e., drop outs)	Nonverbal (MEA)	WCLC Peak‐Picking Algorithm	Lower synchrony at the beginning of therapy associated with premature termination of therapy Higher synchrony linked to lower dropout rates
11. Lutz et al. (2020)	212 Pt 78 Th	AffD AnxD PersD	CBT	—	Overall therapy outcomes (i.e., interpersonal problems and overall symptoms)	Nonverbal (MEA)	WCLC Peak‐Picking Algorithm	Lower synchrony at the beginning of treatment linked to faster improvement in interpersonal problems, which in turn predicted better overall therapy outcomes in both interpersonal problems and overall symptoms
12. Prinz et al. (2020)	175 Pt 57 Th	AffD, AnxD, ED PersD	CBT	Change processes	—	Nonverbal (MEA)	Cross‐correlations within defined time windows	Nonverbal synchrony associated with higher mastery and less resource activation within dyads but showed no significant association with motivational clarification or problem actuation
13. Deres‐Cohen et al. (2021a)	75 dyads	MDD	PDT (SET)	Alliance ruptures	Overall therapy outcomes	Nonverbal (MEA)	Cross‐correlations within defined time windows	Synchrony significantly associated with confrontational ruptures, whereas withdrawal ruptures showed no such association
14. Deres‐Cohen et al. (2021b)	86 Pt 9 Th	MDD	PDT (SET)	Effect of supportive techniques on synchrony	Overall therapy outcomes	Nonverbal (MEA)	Cross‐correlations within defined time windows Polynomial Regression Response Surface Analysis	Greater use of supportive techniques associated with higher levels of synchrony, particularly for patients with lower levels of depression severity and personality disorders No associations for Pts with higher levels of depression severity and personality disorders
15. Nyman‐Salonen et al. (2021)	66 dyads	—	Couple therapy	Therapeutic alliance Effect of Pt′s before sessions well‐being on synchrony	—	Nonverbal (MEA)	SUSY	Pt evaluated the alliance as stronger when there was more body (with respect to head) synchrony in the session For the Th, both head and body synchrony were related to their evaluations of the alliance Higher well‐being at the beginning of sessions predicted higher body (but not head) synchrony within dyads
16. Zimmermann et al. (2021)	16 dyads	Adolescent BPD	PDT (AIT)	—	Overall therapy outcomes	Nonverbal (MEA)	Cross‐correlations within defined time windows	Higher synchrony associated with better overall therapy outcomes in terms of personality functioning Higher synchrony associated with Th's evaluation of Pt′s lower progress ratings
17. Schoenherr et al. (2021)	64 Pt 31 Th	SAD	CBT PDT	Therapeutic alliance	Overall therapy outcomes (i.e., symptoms severity, interpersonal problems and insecure attachment)	Prosody (vocal pitch)	Mixed effects linear models	Higher vocal pitch synchrony was linked to higher symptom severity (social anxiety, avoidance and interpersonal problems) and higher attachment anxiety and avoidance at the end of therapy No effect of vocal pitch synchrony on therapeutic alliance
18. Gernert et al. (2023)	25 Dyads	Not specified diagnosis	CBT	Therapeutic Alliance	Overall Therapy Outcomes	Nonverbal (MEA)	WCLC	Head synchrony negatively associated with Pt′s ratings of therapeutic alliance No associations between synchrony and overall therapy outcomes

Abbreviations: AdjD, adjustment disorder; AffD, affective disorder; AIT, adolescent identity treatment; AnxD, anxiety disorder; BPD, borderline personality disorder; BPT, body‐oriented psychotherapy; CBT, cognitive‐behavioural therapy; CCFs, cross‐correlation functions; ED, eating disorder; MDD, major depressive disorder; MEA, motion energy analysis; n.s., not specified; PDT, psychodynamic therapy; PersD, personality disorder; Pt, patient; SAD, social anxiety disorder; SCZ, schizophrenia; SET, supportive‐expressive therapy; SUSY, surrogate synchrony; Th, therapist; WCLC, windowed cross‐lagged correlation.

**TABLE 3:** Summary of intersubjective interbrain synchrony studies.

Study	Sample	Patient sample features	Intervention	Therapeutic domain		Index	Analysis	Findings
				Process	Outcome			
1. Zhang et al. (2018)	34 dyads	—	Integrative psychotherapy	Therapeutic alliance	—	Interbrain synchrony (fNIRS)	WTC	Increased interbrain synchrony in right TPJ associated with greater Pt′s and Th′s therapeutic alliance
2. Zhang et al. (2019)	30 dyads	—	Integrative psychotherapy	Therapeutic alliance	—	Interbrain synchrony (fNIRS)	WTC	Increased right TPJ interbrain synchrony in experienced Ths, with respect to novice Ths In experienced Ths right TPJ interbrain synchrony associated with greater Pt′s therapeutic alliance Higher Pt‐led interbrain synchrony related to higher goal development only with experienced Ths
3. Lecchi et al. (2020)	14 dyads	—	n.s.	Effect of Th′s mindfulness on synchrony Therapeutic alliance	—	Interbrain synchrony (Dual‐EEG)	PLV	Ths with mindfulness experience associated with higher interbrain synchrony in sessions (respect to Ths without mindfulness experience) High interbrain synchrony correlates with higher rates of Pt′s and Th′s therapeutic alliance
4. Akimoto et al. (2021)	6 dyads	—	PDT (sandplay therapy)	Effect of nonverbal and verbal interaction on synchrony	—	Interbrain synchrony (NIRS)	Spearman's rank‐order correlation coefficients Cross‐spectral analysis	Negative association between interbrain synchrony and lateral PFC and FP during the nonverbal sandplay condition Positive correlation between interbrain synchrony and FP during the verbal interview condition

Abbreviations: EEG, electroencephalography; fNIRS, functional near‐infrared spectroscopy; FP, frontal pole; NIRS, near‐infrared spectroscopy; PDT, psychodynamic therapy; PFC, prefrontal cortex; PLV, phase locking value; Pt, patient; Th, therapist; TPJ, temporoparietal junction; WTC, wavelet transform coherence.

### 2.4 Study Risk of Bias Assessment

Two independent reviewers (LLA and BV) conducted the risk of bias assessment for each study using the *Newcastle‐Ottawa Risk of Bias Scale* (Wells et al. 2013), which evaluates nonrandomized studies based on selection, comparability and outcome assessment. They worked independently, and any discrepancies were resolved through discussion. No automation tools were used in this process. The complete risk of bias assessment is provided in the Supporting Information (Table S1).

## 3 Results

### 3.1 Study Selection and Characteristics

We identified 32 studies, which we subsequently categorized based on the specific index investigated. We then obtained 11 studies related to the interoexteroceptive self (i.e., 9 on EDA, 3 on respiratory activity and 1 on cardiac activity), 18 studies focused on the exteroproprioceptive self (i.e., 15 on nonverbal behaviour and 3 on prosody) and 4 studies examining interbrain synchrony (Figure 3b). Among these, 31 studies investigated a single index, whereasd only one study explored both levels of the self (Gernert et al. 2023). As a result, this study was included in both groups of studies. Finally, different psychotherapeutic interventions were used in the studies reviewed, most notably cognitive‐behavioral therapy (CBT), psychodynamic therapy (PDT) and couple therapy (Figure 3b).

We organized the findings along two core dimensions: *therapeutic process* and *therapeutic outcomes*. The therapeutic process refers to within‐session dynamics between therapist and patient—such as therapeutic alliance, shared emotional experiences and change mechanisms (Gelo et al. 2015)—representing the immediate framework in which therapeutic change begins. Therapeutic outcomes include both *session‐level outcomes* (e.g., emotional shifts and session evaluations) and *overall outcomes* (e.g., symptom reduction and well‐being), reflecting the cumulative impact of these processes over time (Orlinsky et al. 2004).

### 3.2 Risk of Bias in Studies

A structured risk of bias assessment using the Newcastle‐Ottawa Scale indicated high overall methodological quality of the reviewed studies. Most studies scored highly in the domains of case definition and exposure ascertainment, reflecting clear operationalization of synchrony measures and appropriate participant selection. However, moderate risk was frequently observed in the comparability domain, where potential confounding variables (e.g., session content, symptom severity and dyadic characteristics) were not always adequately controlled. In addition, nonresponse rates or dropout data were often insufficiently reported. Of the 32 included studies, 22 achieved the maximum quality rating (8 out of 8), 4 scored 7 out of 8 and 6 received a score of 6 out of 8 (Table S1).

### 3.3 Intersubjective Interoexteroceptive Self Synchrony Studies

#### 3.3.1 Intersubjective Cardiac Synchrony Between Therapist and Patient

**FIGURE 4:**
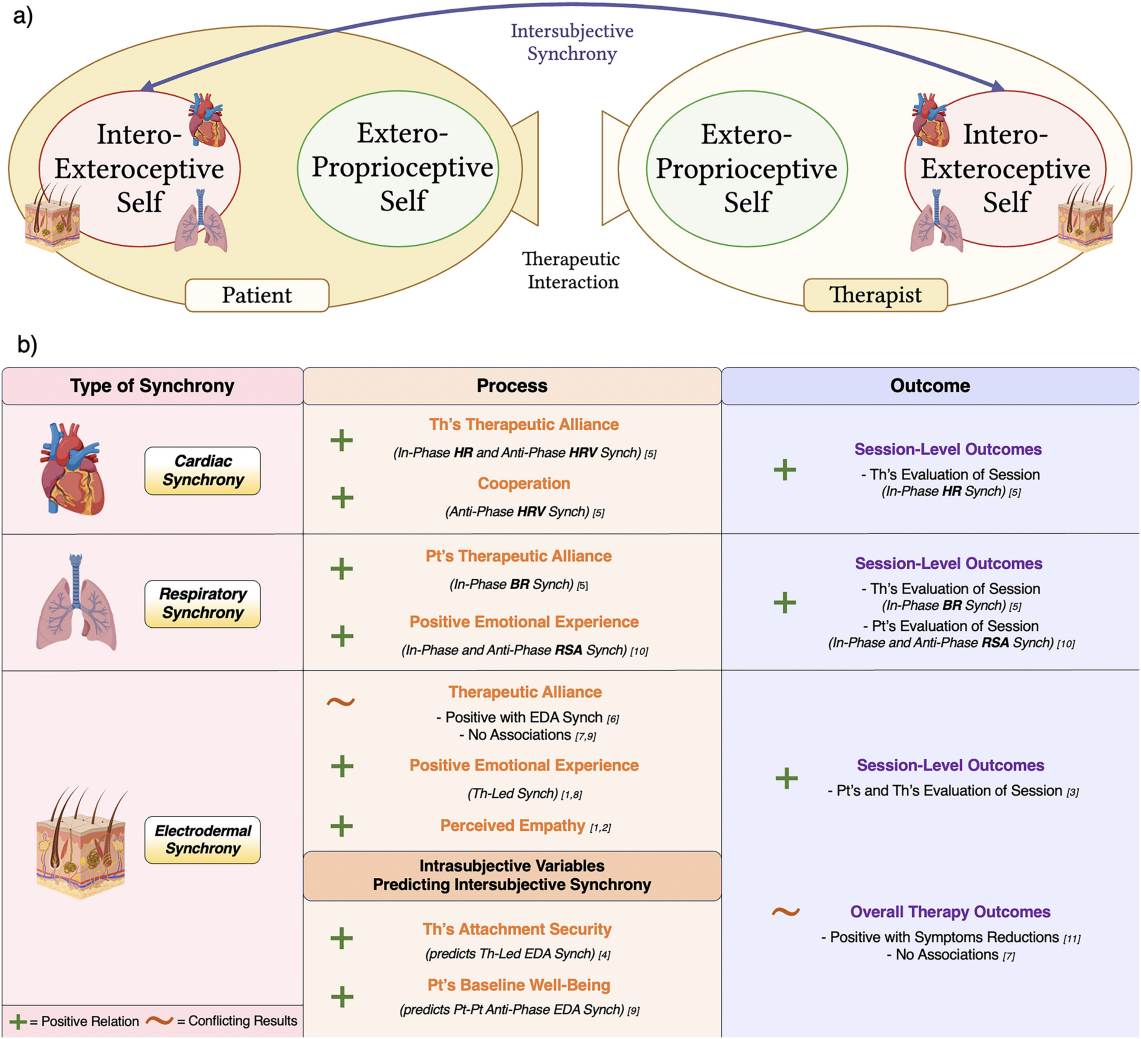
(a) Visual representation of the intersubjective synchrony between therapist's and patient's interoexteroceptive self and (b) a summary of findings of the reviewed studies. Refer to Table 1 to see studies [n]. Abbreviations: BR, breathing rate; EDA, electrodermal activity; HR, heart rate; HRV, heart rate variability; Pt, patient; RSA, respiratory sinus arrhythmia; Th, therapist.

Cardiac synchrony was examined as a marker of therapeutic processes and outcomes in a single study (Table 1 and Figure 4). Tschacher and Meier (2019) monitored heart rate (HR), heart rate variability (HRV) and electrocardiogram (ECG) continuously across four dyads during PDT sessions. Antiphase HR synchrony (inverse cofluctuation) was associated with therapists' ratings of therapeutic alliance, whereas in‐phase HR synchrony (joint HR increase/decrease) was linked to perceived session progress. Similarly, in‐phase HRV synchrony corresponded to higher alliance ratings, whereas antiphase HRV synchrony aligned with therapists' perceptions of client cooperation. No significant associations emerged for ECG synchrony.

#### 3.3.2 Intersubjective Respiratory Synchrony Between Therapist and Patient

Respiratory synchrony has been examined in relation to intersubjective processes and therapeutic outcomes in three studies (Table 1 and Figure 4). Tschacher and Meier (2019) reported that breathing rate synchrony between therapist and patient was positively associated with patients' alliance ratings. Bar‐Kalifa et al. (2023) found that both in‐phase and antiphase respiratory sinus arrhythmia (RSA) synchrony occurred during patients' positive and productive emotional experiences, also predicting higher session satisfaction and perceived therapeutic progress. Similarly, Coutinho et al. (2023), in a couple therapy setting, showed that respiratory synchrony was not uniformly present across sessions but emerged mainly during emotionally connected moments.

#### 3.3.3 Intersubjective Electrodermal Synchrony Between Therapist and Patient

EDA synchrony has been investigated in relation to both therapeutic processes and outcomes across several studies (Table 1 and Figure 4). Marci et al. (2007) and Messina et al. (2013) first demonstrated that higher EDA synchrony was associated with patients' perceptions of greater therapist empathy and more positive socioemotional exchanges. This interpersonal attunement was echoed by Bar‐Kalifa et al. (2019), who found a significant association between EDA synchrony and stronger therapeutic alliance in CBT dyads. In contrast, studies in couple therapy—Tourunen et al. (2020) and Coutinho et al. (2023)—found increased EDA synchrony between patients over time but no consistent link with patient–therapist alliance.

Directionality of synchrony was addressed by Prinz et al. (2021), who reported that therapist‐led EDA synchrony correlated with patients' enhanced emotional experiences (higher contentment, lower anxiety and depression). Similarly, Palmieri et al. (2018a) showed that therapists' priming affected synchrony patterns: Attachment‐security priming led to therapist‐leading synchrony, whereas positive‐affect priming facilitated therapist‐following synchrony. Coutinho et al. (2023) further showed that antiphase EDA synchrony between patients was predicted by presession patient well‐being, underscoring how intrasubjective states influence intersubjective dynamics.

As for outcomes, Gernert et al. (2023) found that greater EDA synchrony predicted symptom reduction in CBT dyads, whereas low or negative synchrony correlated with symptom aggravation. In a tetradic couple therapy setting, Karvonen et al. (2016) reported highest sympathetic synchrony between cotherapists, followed by therapist–patient and then patient–patient dyads, with higher synchrony linked to more positive session evaluations. However, Tourunen et al. (2020) found no significant relation between EDA synchrony and overall outcomes in couple therapy.

#### 3.3.4 Summary of Intersubjective Interoexteroceptive Self Synchrony Findings

Interoexteroceptive self synchrony studies in psychotherapy primarily focused on therapeutic processes rather than outcomes (Table 1 and Figure 4). Cardiac synchrony was associated with higher alliance ratings by therapists, whereas respiratory and EDA synchrony were linked to stronger alliance ratings by patients. However, each modality was examined in isolation, with only one study per index reporting this association, and mixed results for EDA synchrony.

Synchrony across these physiological layers also corresponded to the emotional experience during sessions. RSA and EDA synchrony, particularly the latter, were consistently related to more positive and productive emotional states, especially when the therapist led the synchrony. EDA synchrony also correlated with patients' perceived empathy, whereas cardiac synchrony was linked to perceived cooperation. Additionally, presession intrasubjective traits influenced synchrony: Therapist attachment‐security priming and patient well‐being predicted distinct EDA synchrony patterns, highlighting the influence of individual variables on dyadic dynamics.

All three physiological indices were associated with session‐level outcomes, including higher patient and therapist evaluations. Moreover, higher EDA synchrony was generally linked to symptom reduction, whereas low or negative synchrony correlated with symptom worsening. Yet results for long‐term outcomes remained inconsistent, especially in couple therapy settings. All included studies received the highest risk of bias rating (8/8), except for Tschacher and Meier (2019), which scored 7/8. Effect sizes were not reported.

### 3.4 Intersubjective Exteroproprioceptive Self Synchrony Studies

#### 3.4.1 Intersubjective Prosody Synchrony Between Therapist and Patient

**FIGURE 5:**
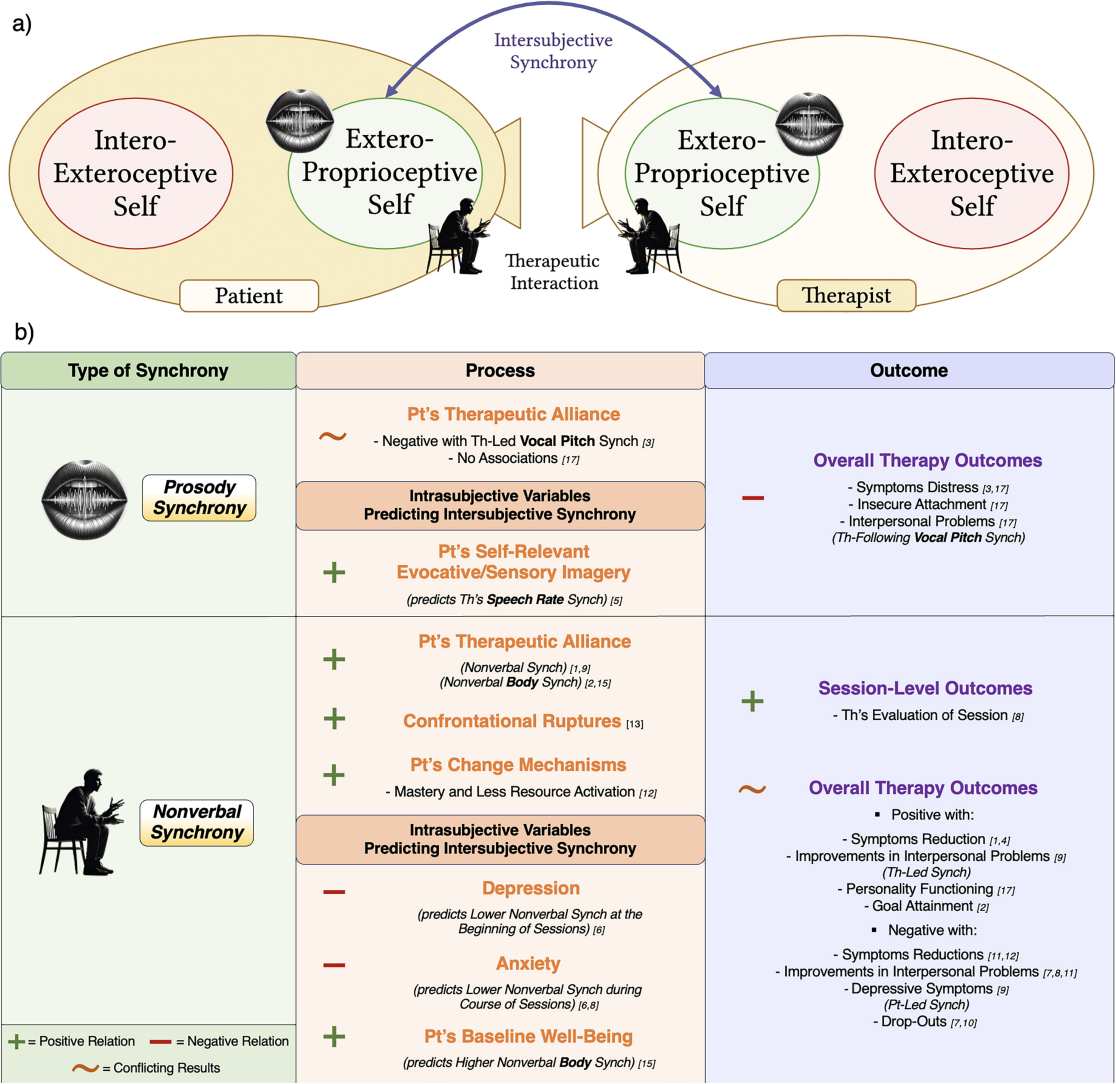
(a) Visual representation of the intersubjective synchrony between therapist's and patient's exteroproprioceptive self and (b) a summary of findings of the reviewed studies. Refer to Table 2 to see studies [n]. Abbreviations: Pt, patient; Th, therapist.

Prosody synchrony, including vocal pitch and speech rate, has been examined for its role in therapeutic processes and outcomes (Table 2 and Figure 5). Reich et al. (2014) and Schoenherr et al. (2021) investigated vocal pitch synchrony in therapist–patient dyads. Higher therapist‐led synchrony was associated with lower patient ratings of the therapeutic alliance (Reich et al. 2014). In the same study, therapist alignment to patient pitch shifts was associated with increased depressive symptoms. Schoenherr et al. (2021), focusing on patients with social anxiety disorder, found that greater vocal pitch synchrony was related to higher symptom severity, including social avoidance and interpersonal problems, as well as to higher attachment anxiety and avoidance.

Rocco et al. (2018) examined speech rate synchrony, showing that higher patient speech rate was linked to more structured discourse, whereas lower speech rate was associated with evocative, imagery‐rich expression. During these slower, emotionally salient phases, therapists matched the patients' speech rate, reflecting moment‐to‐moment synchrony in prosodic dynamics.

#### 3.4.2 Intersubjective Nonverbal Synchrony Between Therapist and Patient

Nonverbal synchrony in psychotherapy has been widely investigated for its association with therapeutic processes and outcomes (Table 2 and Figure 5). Ramseyer and Tschacher (2011) showed that greater synchrony predicted stronger therapeutic alliance and symptom reduction. This link was confirmed by Altmann et al. (2019), who found that early‐session synchrony predicted stronger alliances, with therapist‐led synchrony related to reduced interpersonal problems and patient‐led synchrony associated with worse outcomes. Ramseyer and Tschacher (2014) differentiated head and body synchrony: The former was associated with overall outcomes, the latter with alliance. Similarly, Nyman‐Salonen et al. (2021) found that patients rated stronger alliances when body synchrony exceeded head synchrony, whereas therapists relied on both types.

Other studies reported no consistent link between synchrony and alliance. Paulick et al. (2018b), Ramseyer (2019) and Zimmermann et al. (2021) (adolescents with borderline personality disorder/BPD) found no association. Gernert et al. (2023) observed a negative correlation between head synchrony and alliance ratings, reinforcing previous findings on the primacy of body synchrony (Ramseyer and Tschacher 2014; Nyman‐Salonen et al. 2021). Additionally, Deres‐Cohen et al. (2021a) found synchrony associated with confrontational, but not withdrawal, ruptures.

Regarding therapeutic outcomes, Paulick et al. (2018b) noted that moderate synchrony was linked to improvement, whereas very low or high levels were related to dropout or lack of progress. Schoenherr et al. (2019) confirmed that lower early synchrony predicted early termination. Conversely, Ramseyer (2019) and Lutz et al. (2020) found that lower synchrony predicted faster interpersonal improvements and better outcomes. In contrast, Galbusera et al. (2016) and Zimmermann et al. (2021) found higher synchrony linked to outcome improvements in patients with schizophrenia spectrum and adolescent BPD, respectively. Gernert et al. (2023) found no association between synchrony and overall outcomes.

Some studies examined how presession individual traits affect synchrony. Paulick et al. (2018a) found that depressive patients started with lower synchrony than anxious ones but synchrony increased over time for the former (*d* = 0.35), while remaining stable for the latter (*d* = −0.08). Ramseyer (2019) reported that higher symptom distress at intake predicted lower synchrony throughout treatment. Nyman‐Salonen et al. (2021) found that higher patient well‐being at session start predicted greater body synchrony. Finally, Deres‐Cohen et al. (2021b) found that supportive techniques increased synchrony, particularly in patients with lower depression severity and fewer personality disorders.

#### 3.4.3 Summary of Intersubjective Exteroproprioceptive Self Synchrony Findings

The diverse findings on exteroproprioceptive self synchrony underscore the complexity of prosodic and nonverbal coordination in psychotherapy across different contexts and patient populations (Table 2 and Figure 5). Prosodic synchrony yielded contrasting patterns: Speech rate synchrony played a key role in emotional resonance, as therapists attuned to patients' slower speech during emotionally charged moments. Conversely, vocal pitch synchrony was associated with lower therapeutic alliance and poorer outcomes, especially among anxious or avoidant patients, possibly reflecting maladaptive engagement or heightened emotional distress.

Nonverbal synchrony, particularly body synchrony, consistently emerged as a robust predictor of therapeutic alliance. Therapist‐led synchrony was linked to stronger engagement and positively associated with therapeutic processes, such as mastery strategies and confrontational rupture resolution. In contrast, head synchrony showed weaker or negative associations with alliance ratings.

Regarding outcomes, results were mixed. Although prosodic synchrony was generally linked to poorer outcomes, nonverbal synchrony displayed more variability. Extremely low or high synchrony levels were associated with nonimprovement or dropout, particularly in CBT. Moderate levels appeared most beneficial, whereas higher synchrony predicted better outcomes in patients with psychosis or BPD. Similarly, faster improvement in interpersonal problems was sometimes observed with lower synchrony, suggesting that synchrony may play different roles depending on therapeutic focus and population.

Presession intrasubjective traits influenced synchrony patterns. Depressive patients began with lower synchrony, which increased over therapy, whereas anxious patients remained stable. Higher presession well‐being correlated with greater body synchrony, whereas supportive techniques were also associated with increased synchrony, particularly in patients with lower depression severity and fewer personality disorders.

All cited studies received the highest risk of bias rating (8/8), except for Galbusera et al. (2016), Paulick et al. (2018b), Schoenherr et al. (2019, 2021), Deres‐Cohen et al. (2021a, 2021b), Altmann et al. (2019) and Nyman‐Salonen et al. (2021), which scored 6/8 or 7/8. Moderate effect sizes were only reported by Paulick et al. (2018a).

### 3.5 Intersubjective Interbrain Synchrony Studies

**FIGURE 6:**
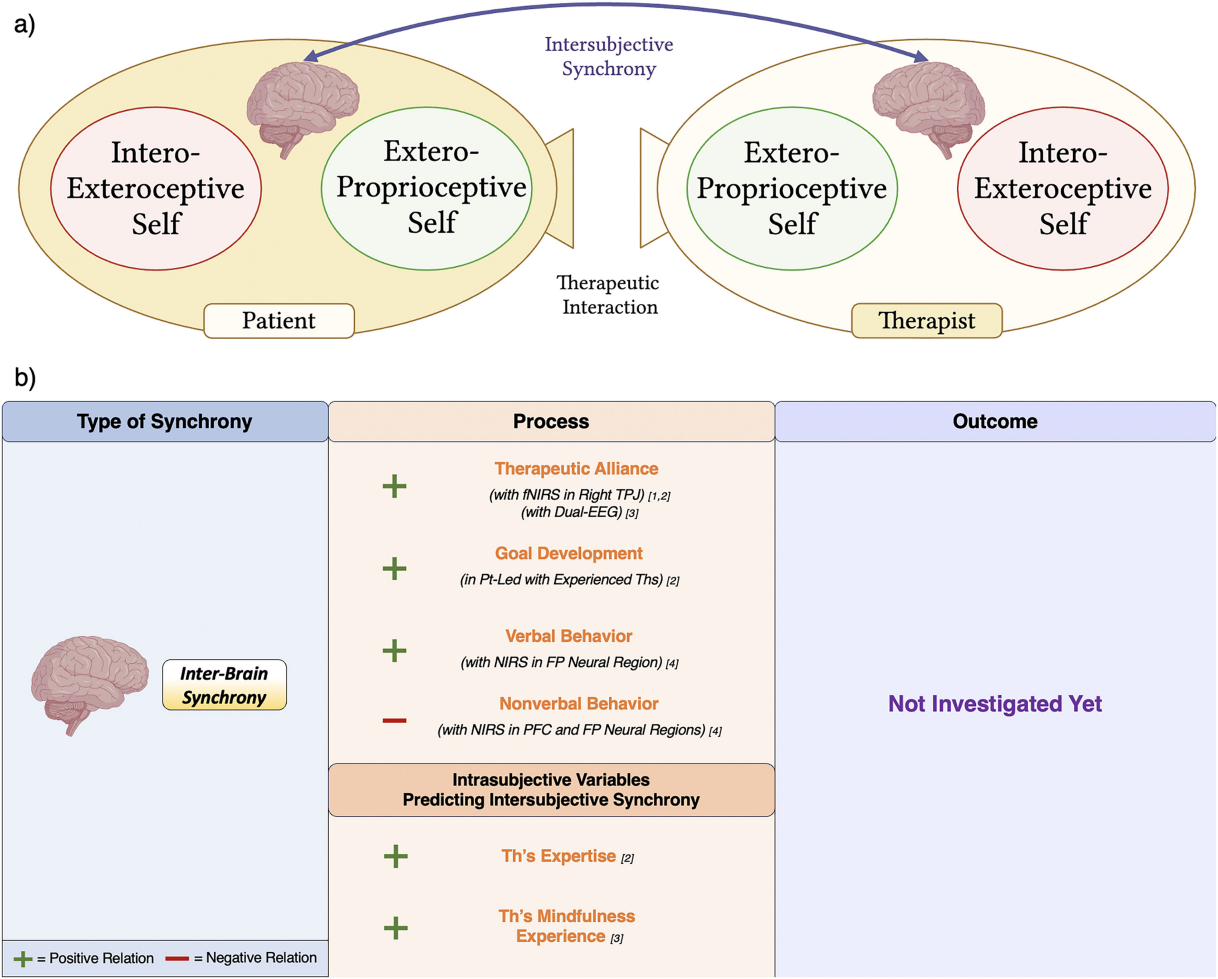
(a) Visual representation of the intersubjective synchrony between therapist's and patient's brain and (b) a summary of findings of the reviewed studies. Refer to Table 3 to see studies [n]. Abbreviations: EEG, electroencephalogram; fNIRS, functional near‐infrared spectroscopy; NIRS, near‐infrared spectroscopy; Pt, patient; Th, therapist.

Interbrain synchrony has recently been investigated in relation to psychotherapeutic processes, particularly the therapeutic alliance (Table 3 and Figure 6). Zhang et al. (2018), using dual‐functional near‐infrared spectroscopy (fNIRS), found greater synchrony in the right temporo‐parietal junction (rTPJ) during therapy sessions compared to casual interactions, with higher synchrony associated with stronger therapeutic alliance. In a follow‐up, Zhang et al. (2019) observed that experienced therapists achieved greater rTPJ synchrony than novices, which corresponded to higher alliance ratings from patients.

Lecchi et al. (2020), using dual‐EEG, found that therapists with mindfulness training exhibited higher interbrain synchrony, which was also linked to a stronger alliance. Notably, synchrony levels were comparable across in‐person and video sessions. Akimoto et al. (2021) applied dual‐NIRS to Sandplay Therapy and observed negative synchrony in the lateral prefrontal cortex (PFC) and frontopolar (FP) regions during nonverbal tasks, shifting to positive synchrony in the FP region during verbal sessions.

#### 3.5.1 Summary of Intersubjective Interbrain Synchrony Findings

Interbrain synchrony studies in psychotherapy revealed significant associations with therapeutic alliance. Stronger synchrony in the right TPJ correlated with better therapeutic alliance, especially for experienced therapists. Moreover, the right TPJ synchrony was more pronounced during therapy rather than casual conversations, confirming its context‐specific nature. In addition, therapists with mindfulness training demonstrated increased interbrain synchrony, which positively related to both patient and therapist alliance assessments. Notably, synchrony levels remained consistent across in‐person and video sessions. Finally, distinct patterns of interbrain synchrony emerged in nonverbal (negative PFC/FP) versus verbal (positive FP) interactions, suggesting distinct neural processes for different therapeutic modalities. No studies investigated the associations of interbrain synchrony and therapeutic outcomes yet. All studies received the highest risk of bias rating (8/8). Effect sizes were not reported.

## 4 Discussion

The present systematic review aimed to investigate the relationship between intersubjective synchrony and therapeutic processes and outcomes across different layers of self‐processing (interoexteroceptive, exteroproprioceptive and interbrain) during the psychotherapeutic interaction between patient and therapist. Intersubjective synchrony is crucial for identifying psychological, biological and behavioural markers that could help personalize psychotherapy by tailoring treatment and synchrony levels to each patient's and therapist's unique predisposition and responsiveness. Additionally, representing an ongoing account of the interaction between patient's and therapist's self, intersubjective synchrony can influence both therapeutic processes and their related short‐ and long‐term outcomes.

### 4.1 Intersubjective Synchrony of the Layers of Self‐Relation to Therapeutic Processes

Therapeutic alliance was the most frequently investigated therapeutic process in relation to intersubjective synchrony among the reviewed studies. It represents a dynamic, multilayered process in which the therapist and the patient collaborate to set goals, agree on tasks and build trust (Gelo et al. 2015), and it is widely recognized as one of the strongest predictors of successful therapy outcomes (Horvath et al. 2011). This process can be understood by exploring how different aspects of self‐processing are involved (i.e., interoexteroceptive, exteroproprioceptive and interbrain).

The therapeutic alliance between patient and therapist was found to be associated with intersubjective synchrony at both interoexteroceptive and exteroproprioceptive levels of self‐processing, as well as with interbrain synchrony. Notably, the ability to develop a stronger alliance was most consistently associated with higher degrees of nonverbal synchrony, that us, at the exteroproprioceptive level. Specifically, higher levels of intersubjective synchrony in nonverbal bodily behaviour (particularly when led by the therapist) were linked to better alliance reported by both patient and therapist (Ramseyer and Tschacher 2011, 2014; Altmann et al. 2019; Nyman‐Salonen et al. 2021). In addition, nonverbal synchrony was associated with specific processes of therapeutic alliance, such as a greater likelihood of engaging in confrontational (rather than withdrawal) ruptures as well as mechanisms of change (like mastery strategies and resource activation) (Prinz et al. 2020; Deres‐Cohen et al. 2021a).

This evidence is further supported by findings from interbrain synchrony studies, which consistently investigated the therapeutic alliance in dyads. They identified the right TPJ as the most activated brain region when the therapist's and patient's brains synchronize over time, engendering a sense of alliance (Zhang et al. 2018, 2019). The TPJ is a key region involved in exteroproprioceptive self‐processing and has been shown to integrate spatial cues and bodily signals from the external environment with one's internal sense of self (Park and Blanke 2019; Qin et al. 2020). This integration supports a coherent body representation in space, which is essential for nonverbal communication and coordinated interaction with others. Additionally, the TPJ plays a central role in theory of mind, that is, the cognitive ability to understand and infer the thoughts, intentions and emotions of others (Frith and Frith 2006). This capacity enables individuals to navigate social interactions by recognizing and responding to the mental states of others, which is crucial for developing mutual understanding and rapport in the therapeutic relationship. Importantly, the inclusion of interbrain synchrony is not only illustrative of neural‐level intersubjective coupling but also conceptually relevant, as it captures the integration between exteroproprioceptive processes and higher order sociocognitive functions—such as mentalizing and empathy—that are central to the therapeutic alliance. Altogether, the involvement of this brain region reinforces the evidence that nonverbal synchronization may be one of the most effective mechanisms for fostering and enhancing the therapeutic alliance within the dyad.

In contrast, studies examining intersubjective synchrony through prosody did not reveal consistent associations with therapeutic alliance. Although one study found no significant relationship between vocal pitch synchrony and alliance in a psychopathological sample (Schoenherr et al. 2021), another reported that therapist‐led pitch synchrony could even have detrimental effects, that is, increased depressive symptoms (Reich et al. 2014). Although most of the reviewed studies focused on vocal pitch, Rocco et al. (2018) investigated speech rate synchrony, finding that therapists tended to match patients' speech rate during emotionally significant moments—suggesting a potential role in enhancing emotional resonance and mutual attunement. Similarly, Imel et al. (2013) found that vocal arousal synchrony was associated with therapist empathy, though this was not replicated by Gaume et al. (2019). Altogether, these findings suggest that prosodic features like speech rate and vocal arousal may be more closely tied to the therapeutic alliance than vocal pitch, highlighting the need for further investigation.

Few studies have examined how interoexteroceptive synchrony, as assessed through physiological markers (such as heart rate, respiration rate and EDA), influences the therapeutic alliance. Only one study reported a positive connection between therapeutic alliance and cardiac and respiratory intersubjective synchrony (Tschacher and Meier 2019). In contrast, three studies investigated the link between the alliance and EDA. Of these, only one found a positive association with therapeutic alliance (Bar‐Kalifa et al. 2019), whereas the other two reported no significant relationship (Tourunen et al. 2020; Coutinho et al. 2023). These mixed results call for caution in drawing conclusions about the role of the therapeutic alliance at the interoexteroceptive layer.

By contrast, interoexteroceptive synchrony has more frequently been linked to the emotional dimension of the therapeutic process. In particular, synchrony in interoexteroceptive measures—especially EDA—has been associated with more positive and therapeutically constructive emotional experiences (Marci et al. 2007; Prinz et al. 2021; Bar‐Kalifa et al. 2023), as well as with an increased patient perception of being empathically listened to by the therapist (Marci et al. 2007; Messina et al. 2013). These results align with theories suggesting that emotions originate from the body's physiological responses, with the neural representation of these states—through ‘somatic markers’—evoking feelings that shape higher layers of self‐processing related to expression/behaviour and cognition (i.e., exteroproprioceptive and mental self) (Craig 2009; Damasio 2010).

One explanation for this functional difference between the two layers of the self may lie in the distinction between their dynamics. Although interoexteroceptive self‐processing dynamics are faster, reflecting rapid, instinctive responses to internal and external stimuli (Craig 2009), exteroproprioceptive self‐processing dynamics are slower, as they involve bodily‐related processes and the integration of the self in space, contributing to a clearer self‐other distinction (Tsakiris 2017). This slower processing allows for a more refined perception of personal boundaries and relational space, essential for distinguishing one's own experiences from those of others. Furthermore, this temporal distinction also corresponds to specific timescales in interoexteroceptive and exteroproprioceptive self‐processing. The former often occurs within milliseconds to seconds (i.e., heart rate, respiration and EDA), allowing for immediate, moment‐to‐moment adjustments to the intersubjective environmental requests (Palumbo et al. 2017). In contrast, the latter (including prosodic and nonverbal cues) typically unfolds over longer timescales (ranging from seconds to minutes) enabling more gradual coordination and alignment in social interactions (Ramseyer and Tschacher 2011; Louwerse et al. 2012). These distinct timescales may thus contribute differently to the layered interaction between the self and its environment, gradually shaping the alliance between therapist and patient over time. This underscores the importance of considering these specific dynamics to better understand each process within the therapeutic exchange.

Altogether, results may suggest that developing a sense of alliance between patient and therapist may require dynamics more closely related to the exteroproprioceptive self, which involves bodily‐based interactions (such as facial expressions, bodily posture and movements) and unfold over longer periods, helping to establish trust and rapport. In contrast, dynamics associated with the interoexteroceptive self may be more involved in immediate emotional reactions and different emotional experiences throughout the therapeutic process. These faster internal physiological responses (like changes in heart rate, breathing rate or EDA) might not directly impact the alliance but play a role in the moment‐to‐moment emotional experience of the therapeutic interaction. Importantly, these faster physiological responses are embedded and thus nested within slower behavioral and social dynamics: Although emotional reactions may fluctuate rapidly, these fast fluctuations are integrated into and contribute to the slower, broader, more gradual development of social and relational dynamics, such as the formation of the therapeutic alliance over time. This resembles the distinction between trait‐ and state‐like alliance (Zilcha‐Mano & Fisher 2022), where trait‐like alliance reflects stable interpersonal characteristics (aligning with exteroproprioceptive dynamics), whereas state‐like alliance embodies evolving, adaptive interactions (resonating with interoexteroceptive dynamics). These varied findings highlight the complex role of the distinct timescales of interoexteroceptive and exteroproprioceptive synchrony in therapy. Achieving optimal therapeutic engagement requires a delicate balance of context‐sensitive synchrony across different timescales. The differing effects of synchrony across patient characteristics and synchrony types underscore the need for further research and more individualized approaches in various therapeutic contexts.

### 4.2 Intersubjective Synchrony of the Layers of Self‐Relation to Therapeutic Outcomes

The impact of intersubjective synchrony across both layers of the self varied depending on whether outcomes were assessed at the session level or across the entire course of therapy. Studies examining interoexteroceptive synchrony have shown that greater alignment in physiological markers such as heart rate, respiration and EDA was associated with more positively evaluated therapy sessions (Karvonen et al. 2016; Tschacher and Meier 2019; Bar‐Kalifa et al. 2023). These findings underscore the relevance of physiological synchrony in supporting real‐time, moment‐to‐moment processes during therapy. In contrast, studies focusing on exteroproprioceptive self showed mixed associations with long‐term overall therapy outcomes. For example, prosodic synchrony—specifically, therapist alignment with the patient's vocal pitch—was associated with poorer outcomes, including increased depressive symptoms (Reich et al. 2014; Schoenherr et al. 2021). Similarly, findings on nonverbal synchrony were mixed. Extremely high or low levels of nonverbal synchrony were both linked to unfavourable outcomes, such as lack of clinical improvement or premature dropout (Paulick et al. 2018b; Ramseyer 2019; Schoenherr et al. 2019; Lutz et al. 2020). Interestingly, optimal levels of nonverbal synchrony appeared to differ across diagnostic groups: individuals with mood, anxiety or eating disorders benefited from moderate levels of synchrony (Paulick et al. 2018b), whereas patients with psychotic symptoms or BPD exhibited better outcomes with higher degrees of synchrony (Galbusera et al. 2016; Zimmermann et al. 2021).

These patterns suggest that the relationship between intersubjective synchrony and therapeutic efficacy is not linear but may depend on the modality of synchrony, the level of outcome assessed and the specific clinical characteristics of the patient population.

### 4.3 Intersubjective Synchrony of the Layers of Self‐Clinical Implications

Beyond its theoretical significance, research on intersubjective synchrony across layers of the self offers practical insights for improving psychotherapeutic practice. In particular, the strong link between exteroproprioceptive synchrony and the quality of the therapeutic alliance suggests that therapists may benefit from training in embodied awareness. Such training would enhance their sensitivity to nonverbal dynamics—such as mirroring gestures, shifts in posture or facial expressions—and help them better track their own bodily responses during sessions. These somatic reactions, often triggered by the patient's affective expressions, can serve as valuable markers of relational dynamics, providing both diagnostic and interventional cues (Palmieri et al. 2018b).

Crucially, embodied training can support therapists in differentiating between their own material and what is being elicited by the patient. This differentiation helps prevent automatic enactments and fosters a reflective, attuned stance. Recognizing moments of physiological or behavioural disconnection—such as a sudden drop in synchrony or a shift in bodily tension—can also aid in timely repair of alliance ruptures, a well‐established predictor of therapeutic success.

Considering these findings, emerging technologies may further support synchrony‐informed practice. For example, wearable biofeedback tools that monitor real‐time physiological indicators such as HRV, respiration rate or EDA could offer therapists insight into the underlying bodily rhythms of the session. When integrated into clinical monitoring, such tools may help identify moments of relational disengagement or excessive arousal, enabling dynamic and responsive adjustments in therapeutic stance or pacing.

Ultimately, integrating synchrony‐informed strategies—both behavioral and physiological—into therapeutic training and practice holds promise for enhancing alliance stability, improving treatment outcomes and advancing precision in psychotherapy.

### 4.4 Synchrony Connects Intersubjective and Intrasubjective Dynamics in Psychotherapy—Do We Need a Spatiotemporally Informed Approach?

**FIGURE 7:**
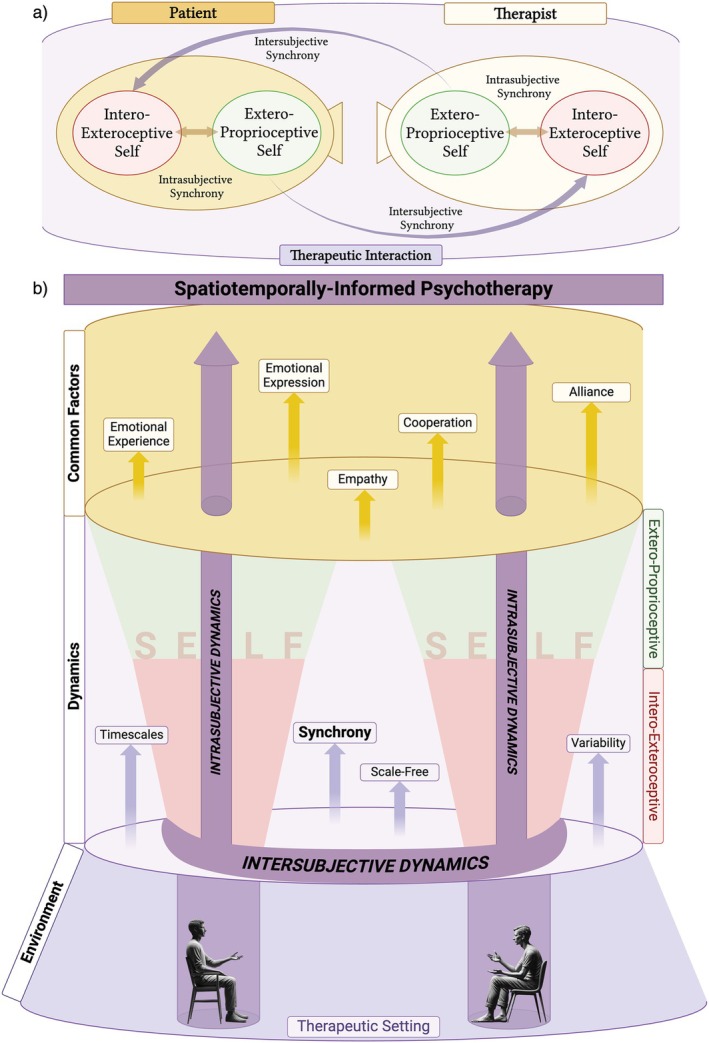
(a) Visual diagram illustrating the dynamic feedback loop of synchrony in a therapeutic interaction between patient and therapist. The therapist's exteroproprioceptive self (nonverbal and prosodic cues) influences the patient's interoexteroceptive self (physiological responses) (purple arrows), prompting changes in the patient's own exteroproprioceptive self (yellow arrows). This, in turn, affects the therapist's interoexteroceptive self and so on, creating a continuous cycle of mutual influence. (b) A conceptual model of the self and its dynamics in psychotherapy. The model illustrates the hierarchical structure of psychotherapeutic factors, from the foundational self and its dynamics to common factors of the relationship. It emphasizes the role of intrasubjective and intersubjective dynamics in shaping common factors of the therapeutic relationship. Created with BioRender.com.

The reviewed studies highlighted the need to thoroughly investigate degrees of intersubjective synchrony to achieve more accurate therapeutic processes and outcomes. This requires considering that existing studies have primarily investigated synchrony between corresponding layers of the self, sharing similar dynamics. Future research could investigate how different layers with distinct dynamics influence each other in a therapeutic setting. For example, it would be useful to explore how the slower dynamics of the exteroproprioceptive self might affect the faster dynamics of the interoexteroceptive self and vice versa. In a therapeutic interaction, the therapist's exteroproprioceptive layer (including prosody and nonverbal behaviour) can affect the patient's interoexteroceptive self‐processing (involving physiological responses like heart rate and respiration). These changes may in turn elicit emotional and behavioral reactions in the patient, which can then reverberate back onto the therapist's own interoexteroceptive processes. What emerges is a continuous and dynamic feedback loop, where each participant's interoexteroceptive and exteroproprioceptive dynamics influence the other's experience of the relationship (Figure 7a). This mutual interaction is crucially reflected by somatic countertransference, where the therapist physically senses and reacts to the patient's emotional state, adapting to meet the patient's needs (Palmieri et al. 2018b). Moreover, this supports the core theoretical claim that the self is not a static entity but a dynamic and relational process shaped by spatiotemporal patterns of interaction (Northoff and Scalabrini 2021). Synchrony becomes the mechanism through which these relational dynamics are enacted in the here‐and‐now of the session, allowing the patient's self to be gradually reorganized.

This continuous feedback loop has been validated by a Kuramoto model for self‐other integration, which was applied to nonclinical intersubjective synchronization tasks (Heggli et al. 2019). This model demonstrated that degrees of synchrony between individuals involves balancing intrasubjective and intersubjective dynamics, supporting the idea that intersubjective synchrony can drive changes in intrasubjective synchrony, which, in turn, can affect the intersubjective process. In the therapeutic exchange, this might mean that when therapist and patient achieve physiological or behavioural synchrony, it can help reorganize the patient's internal state, making them feel more emotionally regulated and connected. As a result, the patient may find it easier not only to engage more deeply in the therapeutic relationship but also to form and maintain more attuned relationships outside of therapy. Findings from the reviewed studies also align with this, showing that intrasubjective traits prior to the session can significantly influence the degree of intersubjective synchrony during therapy. From the therapist perspective, it has been shown that when therapists were primed with attachment‐security inputs prior to sessions, they often led the synchrony, resulting in higher EDA synchrony when anticipating the patient's responses (Palmieri et al. 2018a). Additionally, intrasubjective traits like expertise and mindfulness training were observed to be strongly influential in shaping better therapeutic alliance within dyads at the neural level (Zhang et al. 2019; Lecchi et al. 2020).

On the other hand, reviewed studies on patients' presession intrasubjective traits revealed that patients' psychological and physical well‐being before sessions can affect intersubjective synchrony in EDA and nonverbal behaviour, also correlating with therapeutic outcomes (Coutinho et al. 2023; Nyman‐Salonen et al. 2021). Furthermore, Paulick et al. (2018a) observed that synchrony patterns varied by patient condition: Initially, depressive patients displayed lower synchrony compared to anxious patients, but as therapy progressed, nonverbal synchrony increased in depressive patients, whereas anxious patients maintained the same synchrony levels as at the beginning. In accordance, Ramseyer (2019) noted that patients entering therapy with higher symptom distress showed lower overall intersubjective synchrony during the therapy course. This suggests that intrasubjective psychophysiological states can strongly impact the patient's biological tendency to synchronize with the therapist throughout therapy, also affecting subsequent outcomes. However, studies examining the influence of intrasubjective traits on intersubjective synchrony have primarily relied on self‐reported questionnaires to assess psychopathological traits (e.g., depression and anxiety) and well‐being, leaving the underlying biobehavioural dynamics unexamined.

Future research may explore how neuronal, physiological and behavioural markers reflect patient and therapist intrasubjective dynamics. Using advanced statistical methods to analyse alignment in cardiac, respiratory, nonverbal and brain activity (i.e., Mahmoodi et al. 2023) may reveal connections between these markers and specific behavioural outcomes, opening new possibilities for understanding therapeutic interactions. Through such analyses, it could be possible to explore intrasubjective synchrony between interoexteroceptive and exteroproprioceptive layers for both patient and therapist, offering a biobehavioural context to understand how this impacts the interaction and how it can be mutually modified. These studies could significantly advance personalized therapeutic interventions by identifying key dynamics of therapeutic processes and change, also enhancing a more functional patient‐therapist matching.

In this context, dysfunctional intersubjective and intrasubjective patterns of the patient can be gradually reshaped through the therapist's attuned presence and relational engagement, which introduces emotional and behavioural dynamics that differ from those the patient has typically experienced in past relationships. These new relational experiences—grounded in synchrony, safety and regulation—can serve as corrective emotional experiences (Alexander and French 1946; Guidano 1991), allowing the patient to encounter interpersonal situations in which their emotional states are not ignored, dismissed or escalated but instead recognized, regulated and coprocessed. For example, a therapist's calm prosody, responsive posture or steady physiological state can help modulate the patient's arousal, enabling them to feel more emotionally safe and connected in the moment. Over time, these repeated episodes of coregulation may allow the patient to internalize more stable affective rhythms and develop a more coherent and flexible sense of self. In this way, the therapeutic relationship becomes a space where previously maladaptive self‐patterns are not only understood but also transformed through embodied engagement, giving rise to novel self‐other dynamics that can extend beyond therapy and into the patient's broader relational world. Ultimately, these dynamics give shape to what are often referred to as the ‘common factors of the therapeutic relationship’ (Figure 7b) (Wampold 2015; Cuijpers et al. 2019). These include general elements of the interaction involving the self, such as empathy, alliance, cooperation, and emotional experiences and expressions, which are crucial for therapeutic success across different approaches (Rosenzweig 1936; Luborsky et al. 2002).

Together, these findings highlight the value of a dynamically—and ultimately spatiotemporally—informed approach to psychotherapy, one that prioritizes the self and its embodied dynamics over specific techniques or theoretical orientations. Such an approach suggests that therapeutic change may be primarily driven by relational and process‐based factors grounded in self‐related synchrony. This view aligns with emerging models that emphasize the central role of spatiotemporal self‐dynamics in both psychopathology and psychotherapy (Northoff and Scalabrini 2021). Rather than focusing exclusively on symptom reduction or cognitive content, a spatiotemporally informed psychotherapy would aim to support the reorganization of the self as enacted through multilevel synchrony within the therapeutic relationship.

### 4.5 Limitations of the Systematic Review

It is important to note that a key limitation of this systematic review lies in the substantial heterogeneity across studies in terms of experimental design, operationalization of synchrony and data analysis strategies. This diversity limits the direct comparability of findings and the ability to draw generalizable conclusions. Moreover, most of the studies did not report standardized effect sizes, which constrains the capacity to quantitatively synthesize the strength of associations between synchrony and clinical outcomes. Future work would benefit from the adoption of more consistent methodological standards and transparent reporting practices to enhance cumulative evidence in this emerging field.

## 5 Conclusion

This systematic review underscores the crucial importance of considering the self in psychotherapy research, particularly in understanding the layered dynamics of interoexteroceptive and exteroproprioceptive synchrony. Effective therapeutic engagement relies on context‐sensitive synchrony at different layers through intersubjective interaction for a self‐reorganization of the patients' dynamics. By recognizing how the self functions across different timescales—where faster interoexteroceptive synchrony facilitates moment‐to‐moment emotional attunement and slower exteroproprioceptive synchrony supports trust and alliance‐building—research can better illuminate the mechanisms shaping therapeutic processes and outcomes. Future studies should further investigate self‐related dynamics to identify biobehavioural markers that guide precision psychotherapy, fostering tailored interventions and promoting adaptive, long‐term therapeutic change.

## 6 Glossary

*Interoexteroceptive self*: the most basic layer of self‐processing involving the integration of one's own internal physiological signals (cardiac, respiratory and electrodermal activity).

E*xteroproprioceptive self*: self‐processing focusing on external stimuli (such as prosodic and nonverbal cues) representing the expression/manifestation of both layers in the therapeutic setting.

*Intrasubjective dynamics*: internal processes within each individual's self, encompassing both interoexteroceptive and exteroproprioceptive layers of self and their interaction.

*Intersubjective dynamics*: the reciprocal influence between a patient's and therapist's layers of self, allowing for alignment and mutual understanding in the therapeutic relationship.

*Self‐organization*: the spontaneous process through which a system arranges its internal components to maintain coherence, stability and functionality without requiring external control.

*Self‐reorganization*: the therapeutic restoration of the dynamics between interoexteroceptive and exteroproprioceptive layers of the self (disrupted by relational trauma) through a novel intersubjective relational context provided by therapy.

## Author Contributions

L.L.A., B.V., A.S. and G.N. shared responsibility for the conceptualization of this study. L.L.A. wrote the manuscript. L.L.A. and B.V. collected data. F.G., G.C., V.R., A.S. and G.N. provided critical feedback and helped revise and shape the manuscript. All authors have read and approved the final manuscript.

## Conflicts of Interest

The authors declare no conflicts of interest.

## Supporting information


**Table S1** Risk of bias of the examined studies through Newcastle‐Ottawa Risk of Bias Scale.

## Data Availability

All data used in the analysis are publicly available in the original publications, which are accessible through academic journals and databases. No new data were created or analysed in this study.
