# Supplementary material for: The Self and Its Intersubjective Synchrony in Psychotherapy: A Systematic Review
Source: Clin Psychol Psychother. 2025 Jul 1;32(4):e70110. doi: 10.1002/cpp.70110 (PMC12211550; doi:10.1002/cpp.70110)
Supplement: Supplementary file 1 — Table S1 Risk of bias of the examined studies through Newcastle‐Ottawa Risk of Bias Scale. [file CPP-32-e70110-s001.docx]

**Supplementary Table 1.** Risk of bias of the examined studies through Newcastle-Ottawa Risk of Bias Scale.

| ***Newcastle-Ottawa Risk of Bias Scale*** | | | | | | | | | |
| --- | --- | --- | --- | --- | --- | --- | --- | --- | --- |
| *Studies* | *Selection* | | | | *Comparability* | *Exposure* | | | **Total Quality score** |
| **Author, year** | **Is the Case Definition Adequate?** | **Representativeness of the Cases** | **Selection of Controls** | **Definition of Controls** | **Comparability of cases and controls** | **Ascertainment of exposure** | **Same method of ascertainment for cases and controls** | **Non-Response rate** |  |
| *Intersubjective Intero-Exteroceptive Synchrony studies* | | | | | | | | | |
| ***1****. Marci et al., 2007* | 1 | 1 | 1 | 1 | 1 | 1 | 1 | 1 | 8 |
| ***2****. Messina et al., 2013* | 1 | 1 | 1 | 1 | 1 | 1 | 1 | 1 | 8 |
| ***3****. Karvonen et al., 2016* | 1 | 1 | 1 | 1 | 1 | 1 | 1 | 1 | 8 |
| ***4****. Palmieri et al., 2018* | 1 | 1 | 1 | 1 | 1 | 1 | 1 | 1 | 8 |
| ***5****. Tschacher & Maier, 2019* | 1 | 0 | 1 | 1 | 1 | 1 | 1 | 1 | 7 |
| ***6****. Bar-Kalifa et al., 2019* | 1 | 1 | 1 | 1 | 1 | 1 | 1 | 1 | 8 |
| ***7****. Tourunen et al., 2020* | 1 | 1 | 1 | 1 | 1 | 1 | 1 | 1 | 8 |
| ***8****. Prinz et al., 2021* | 1 | 1 | 1 | 1 | 1 | 1 | 1 | 1 | 8 |
| ***9****. Coutinho et al., 2023* | 1 | 1 | 1 | 1 | 1 | 1 | 1 | 1 | 8 |
| ***10****. Bar-Kalifa et al., 2023* | 1 | 1 | 1 | 1 | 1 | 1 | 1 | 1 | 8 |
| ***11****. Gernert et al., 2023* | 1 | 1 | 1 | 1 | 1 | 1 | 1 | 1 | 8 |
| *Intersubjective Extero-Proprioceptive Synchrony studies* | | | | | | | | | |
| ***1****. Ramseyer & Tschacher, 2011* | 1 | 1 | 1 | 1 | 1 | 1 | 1 | 1 | 8 |
| ***2****. Ramseyer & Tschacher, 2014* | 1 | 1 | 1 | 1 | 1 | 1 | 1 | 1 | 8 |
| ***3****. Reich et al., 2014* | 1 | 1 | 1 | 1 | 1 | 1 | 1 | 1 | 8 |
| ***4****. Galbusera et al., 2016* | 1 | 1 | 1 | 1 | 1 | 1 | 1 | 0 | 7 |
| ***5****. Rocco et al., 2018* | 1 | 1 | 1 | 1 | 1 | 1 | 1 | 1 | 8 |
| ***6****. Paulick et al., 2018a* | 1 | 1 | 1 | 1 | 1 | 1 | 1 | 1 | 8 |
| ***7****. Paulick et al., 2018b* | 1 | 1 | 1 | 1 | 0 | 1 | 1 | 0 | 6 |
| ***8****. Ramseyer, 2019* | 1 | 1 | 1 | 1 | 1 | 1 | 1 | 1 | 8 |
| ***9****. Altmann et al., 2019* | 1 | 0 | 1 | 1 | 1 | 1 | 1 | 0 | 6 |
| ***10****. Schoenherr et al., 2019* | 1 | 1 | 1 | 1 | 0 | 1 | 1 | 0 | 6 |
| ***11****. Lutz et al., 2020* | 1 | 1 | 1 | 1 | 1 | 1 | 1 | 1 | 8 |
| ***12****. Prinz et al., 2020* | 1 | 1 | 1 | 1 | 1 | 1 | 1 | 1 | 8 |
| ***13****. Deres-Cohen et al., 2021a* | 1 | 1 | 1 | 1 | 0 | 1 | 1 | 0 | 6 |
| ***14****. Deres-Cohen et al., 2021b* | 1 | 1 | 1 | 1 | 0 | 1 | 1 | 0 | 6 |
| ***15****. Nyman‐Salonen et al., 2021* | 1 | 1 | 1 | 1 | 1 | 1 | 1 | 0 | 7 |
| ***16****. Zimmermann et al., 2021* | 1 | 1 | 1 | 1 | 1 | 1 | 1 | 1 | 8 |
| ***17****. Schoenherr et al., 2021* | 1 | 1 | 1 | 1 | 0 | 1 | 1 | 0 | 6 |
| ***18****. Gernert et al., 2023* | 1 | 1 | 1 | 1 | 1 | 1 | 1 | 1 | 8 |
| *Inter-Brain Synchrony studies* | | | | | | | | | |
| ***1****. Zhang et al., 2018* | 1 | 1 | 1 | 1 | 1 | 1 | 1 | 1 | 8 |
| ***2****. Zhang et al., 2019* | 1 | 1 | 1 | 1 | 1 | 1 | 1 | 1 | 8 |
| ***3****. Lecchi et al., 2020* | 1 | 1 | 1 | 1 | 1 | 1 | 1 | 1 | 8 |
| ***4****. Akimoto et al., 2021* | 1 | 0 | 1 | 1 | 1 | 1 | 1 | 1 | 7 |
